# Three-fold utilization of supplementary information for mean estimation under median ranked set sampling scheme

**DOI:** 10.1371/journal.pone.0276514

**Published:** 2022-10-24

**Authors:** Usman Shahzad, Ishfaq Ahmad, Ibrahim Mufrah Almanjahie, Amer Ibrahim Al-Omari

**Affiliations:** 1 Department of Mathematics and Statistics, International Islamic University, Islamabad, Pakistan; 2 Department of Mathematics and Statistics - PMAS-Arid Agriculture University, Rawalpindi, Pakistan; 3 Department of Mathematics, College of Science, King Khalid University, Abha, Saudi Arabia; 4 Statistical Research and Studies Support Unit, King Khalid University, Abha, Saudi Arabia; 5 Department of Mathematics, Faculty of Science, Al al-Bayt University, Mafraq, Jordan; Amity University - Lucknow Campus, INDIA

## Abstract

Ranked set sampling (RSS) has created a broad interest among researchers and it is still a unique research topic. It has at long last begun to find its way into practical applications beyond its initial horticultural based birth in the fundamental paper by McIntyre in the nineteenth century. One of the extensions of RSS is median ranked set sampling (MRSS). MRSS is a sampling procedure normally utilized when measuring the variable of interest is troublesome or expensive, whereas it might be easy to rank the units using an inexpensive sorting criterion. Several researchers introduced ratio, regression, exponential, and difference type estimators for mean estimation under the MRSS design. In this paper, we propose three new mean estimators under the MRSS scheme. Our idea is based on three-fold utilization of supplementary information. Specifically, we utilize the ranks and second raw moments of the supplementary information and the original values of the supplementary variable. The appropriateness of the proposed group of estimators is demonstrated in light of both real and artificial data sets based on the Monte-Carlo simulation. Additionally, the performance comparison is also conducted regarding the reviewed families of estimators. The results are empowered and the predominant execution of the proposed group of estimators is seen throughout the paper.

## 1 Introduction

The ranked set sampling (RSS) design was primarily developed by [1] as an effective inspection plan for estimating the population parameters, i.e., the mean of field and scrounge yields. This inspection plan is appropriate in circumstances where the ranking of perceptions is effectively dependent on a supplementary variable, which is linearly related to the subject variable or any reasonable technique. The RSS has wide applications in numerous logical issues, particularly in natural and environmental examinations where the principal concentration is on practical and productive sampling/inspection techniques. For instance, assume that the agency of environmental protection needs to guarantee that the fuel stations in certain metropolitan territories are distributing gas according to air clean guidelines. It is quite possible the compound parameters of fuel can be effectively ranked right after the assortment at the fuel siphon by some rough field strategies being modest and simple. While carrying the sample units to the research center and utilizing certain lab procedures to gauge its compound parameters is costly. For more details about RSS and its applications, see, for example, [2, 3] who suggested the median ranked set sampling (MRSS) for estimating the population mean. The MRSS consists of the following steps:

**Step 1**: Select *n* random samples, each of size *n* units from the population and rank the units within each sample with respect to the variable of interest.
**Step 2**: If the sample size *n* is odd, then from each sample, select for measurement of the $\left(\frac{n+1}{2}\right)$ *th* smallest rank (the median of the sample). If the sample size *n* is even, then select for measurement of the $\left(\frac{n}{2}\right)$ *th* smallest rank from the first $\frac{n}{2}$ samples, and the $\left(\frac{n+2}{2}\right)$ *th* smallest rank from the second $\frac{n}{2}$ samples. The cycle can be repeated *m* times if needed to obtain a sample of size *nm*.

[4, 5] suggested multistage median ranked set sampling for estimating the population mean and median, respectively. [6] considered the modified ratio estimator for the population mean using double MRSS. For more details of RSS and its applications, see [7–17].

[12] considered the median ranked set sampling scheme and built ratio-type estimators based on known quartiles of supplementary information. [18] extended the work of [12] and suggested new families of estimators. These families also contain known mean and quartiles of supplementary information. In this paper, some new estimators for the population mean of the random variable *Y* are proposed under MRSS scheme. The basic idea is when there exists an adequate relationship (sufficient correlation) between (*Y*, *X*), the ranks and second raw moment of the supplementary variable are also correlated with the variate of interest. In light of this reality, we will utilize three forms, i.e., (the original values, ranks and the second raw moment) of a single supplementary variable *X*. The proposed three-fold utilization of the supplementary variable will provide enhancement in the estimation of population mean *μ*_*y*_. So, we build some estimators for estimating *μ*_*y*_ which not only includes the original values of the auxiliary variable *X* but also on the second raw moment and ranks of the supplementary variable. The appropriateness of the recommendation is additionally shown empirically by utilizing real and artificial populations. The execution assessment uncovers the prevalent presentation of the proposed family in contrast with [12, 18] group of estimators.

The rest of the paper is composed of pursues. In Section 1.1, we present documentation (notations) and a concise review of the estimators utilized in MRSS when a supplementary variable is accessible. For every estimator, the theoretical properties of order *n*^−1^ are derived. In Section 2, we extend the idea of [12, 18], in light of three-fold utilization of supplementary information of a single auxiliary variable, and propose three new estimators for estimating *μ*_*y*_ under MRSS with their theoretical properties. In Section 3.1, we perform a reenactment (simulation) study to look at, under changed situations, the productivity of the proposed estimator with that of other focused estimators present in the literature. In Section 3.2, the productivity of the estimator is researched by utilizing a real-life data set. Finally, the conclusion is provided in Section 4.

### 1.1 Preliminaries with respect to median ranked set sampling

Suppose a median ranked set sample of size *n* is drawn from a finite population *ω*, consisting of *N* units. Let *Y* and *X* are study and auxiliary variables, respectively. Further, let us assume that *Z* and *V* representing; the ranks and squared of each value of the supplementary variable, respectively.

Let (*X*_*i*(1)_, *Z*_*i*[1]_, *Y*_*i*[1]_, *V*_*i*[1]_), (*X*_*i*(2)_, *Z*_*i*[2]_, *Y*_*i*[2]_, *V*_*i*[2]_), …, (*X*_*i*(*n*)_, *Z*_*i*[*n*]_, *Y*_*i*[*n*]_, *V*_*i*[*n*]_) be the order statistics of *X*_*i*1_, *X*_*i*2_, …, *X*_*in*_ and the judgment order of *Z*_*i*1_, *Z*_*i*2_, …, *Z*_*in*_; *Y*_*i*1_, *Y*_*i*2_, …, *Y*_*in*_; *V*_*i*1_, *V*_*i*2_, …, *V*_*in*_ for (*i* = 1, 2, …, *n*), where (.) and [.] indicate that the ranking of *X* is perfect and the ranking of *Y* has error. For odd and even sample sizes, the units measured using MRSS are denoted by MRSSO and MRSSE, respectively.

For odd sample size, let $\left(X_{1(\frac{1+n}{2})},Z_{1[\frac{1+n}{2}]},Y_{1[\frac{1+n}{2}]},V_{1[\frac{1+n}{2}]}\right)$, $(X_{2(\frac{1+n}{2})},Z_{2[\frac{1+n}{2}]},Y_{2[\frac{1+n}{2}]},V_{2[\frac{1+n}{2}]}),\ldots$, $(X_{n(\frac{1+n}{2})},Z_{n[\frac{1+n}{2}]},Y_{n[\frac{1+n}{2}]},V_{n[\frac{1+n}{2}]})$ be the observed units by MRSSO. ${\bar{x}}_{MRSSO}=\frac{1}{n}\sum_{i=1}^{n}X_{i(\frac{1+n}{2})}$, ${\bar{z}}_{MRSSO}=\frac{1}{n}\sum_{i=1}^{n}Z_{i[\frac{1+n}{2}]}$, ${\bar{y}}_{MRSSO}=\frac{1}{n}\sum_{i=1}^{n}Y_{i[\frac{1+n}{2}]}$ and ${\bar{v}}_{MRSSO}=\frac{1}{n}\sum_{i=1}^{n}V_{i[\frac{1+n}{2}]}$ be the MRSS mean of *X*, *Z*, *Y* and *V*, respectively. Further, $Var\left({\bar{x}}_{MRSSO}\right)=\lambda_{\left(O\right)}\sigma_{x(\frac{1+n}{2})}^{2}$, $Var\left({\bar{z}}_{MRSSO}\right)=\lambda_{\left(O\right)}\sigma_{z[\frac{1+n}{2}]}^{2}$ and $Var\left({\bar{y}}_{MRSSO}\right)=\lambda_{\left(O\right)}\sigma_{y[\frac{1+n}{2}]}^{2}$, $Var\left({\bar{v}}_{MRSSO}\right)=\lambda_{\left(O\right)}\sigma_{v[\frac{1+n}{2}]}^{2}$ are the variances of ${\bar{x}}_{MRSSO}$, ${\bar{z}}_{MRSSO}$, ${\bar{y}}_{MRSSO}$ and ${\bar{v}}_{MRSSO}$, respectively, where $\lambda_{\left(O\right)}=\frac{1}{n}$.

For even sample size, let $\left(X_{1(\frac{n}{2})},Z_{1[\frac{n}{2}]},Y_{1[\frac{n}{2}]},V_{1[\frac{n}{2}]}\right),\left(X_{2(\frac{n}{2})},Z_{2[\frac{n}{2}]},Y_{2[\frac{n}{2}]},V_{2[\frac{n}{2}]}\right),\ldots ,\left(X_{\frac{n}{2}\left(\frac{n}{2}\right)},Z_{\frac{n}{2}\left[\frac{n}{2}\right]},Y_{\frac{n}{2}\left[\frac{n}{2}\right]},V_{\frac{n}{2}\left[\frac{n}{2}\right]}\right),\left(X_{\frac{2+n}{2}\left(\frac{2+n}{2}\right)},Z_{\frac{2+n}{2}\left[\frac{2+n}{2}\right]},Y_{\frac{2+n}{2}\left[\frac{2+n}{2}\right]},V_{\frac{2+n}{2}\left[\frac{2+n}{2}\right]}\right),(X_{\frac{4+n}{2}\left(\frac{2+n}{2}\right)},Z_{\frac{4+n}{2}\left[\frac{2+n}{2}\right]},Y_{\frac{4+n}{2}\left[\frac{2+n}{2}\right]},V_{\frac{4+n}{2}\left[\frac{2+n}{2}\right]}),\ldots ,\left(X_{n(\frac{n}{2})},Z_{n[\frac{n}{2}]},Y_{n[\frac{n}{2}]},Z_{n[\frac{n}{2}]}\right)$ denote the observed units by MRSSE. Further

$$\begin{array}{c} {\bar{x}}_{MRSSE}=\frac{1}{n}\left(\sum_{i=1}^{\frac{n}{2}}X_{i(\frac{n}{2})}+\sum_{i=\frac{2+n}{2}}^{n}X_{i(\frac{2+n}{2})}\right),\;{\bar{z}}_{MRSSE}=\frac{1}{n}\left(\sum_{i=1}^{\frac{n}{2}}Z_{i[\frac{n}{2}]}+\sum_{i=\frac{2+n}{2}}^{n}Z_{i[\frac{2+n}{2}]}\right), \end{array}$$

$$\begin{array}{c} {\bar{y}}_{MRSSE}=\frac{1}{n}\left(\sum_{i=1}^{\frac{n}{2}}Y_{i[\frac{n}{2}]}+\sum_{i=\frac{2+n}{2}}^{n}Y_{i[\frac{2+n}{2}]}\right)\;{\bar{v}}_{MRSSE}=\frac{1}{n}\left(\sum_{i=1}^{\frac{n}{2}}V_{i[\frac{n}{2}]}+\sum_{i=\frac{2+n}{2}}^{n}V_{i[\frac{2+n}{2}]}\right), \end{array}$$

are the MRSS mean of *X*, *Z*, *Y* and *V*, respectively. Also, $Var\left({\bar{x}}_{MRSSE}\right)=\lambda_{\left(E\right)}\left(\sigma_{x(\frac{n}{2})}^{2}+\sigma_{x(\frac{2+n}{2})}^{2}\right)$, $Var\left({\bar{z}}_{MRSSE}\right)=\lambda_{\left(E\right)}\left(\sigma_{z[\frac{n}{2}]}^{2}+\sigma_{z[\frac{2+n}{2}]}^{2}\right)$, $Var\left({\bar{y}}_{MRSSE}\right)=\lambda_{\left(E\right)}\left(\sigma_{y[\frac{n}{2}]}^{2}+\sigma_{y[\frac{2+n}{2}]}^{2}\right)$ and $Var\left({\bar{v}}_{MRSSE}\right)=\lambda_{\left(E\right)}\left(\sigma_{V[\frac{n}{2}]}^{2}+\sigma_{V[\frac{2+n}{2}]}^{2}\right)$ are the variances of ${\bar{x}}_{MRSSE},{\bar{z}}_{MRSSE}$, ${\bar{y}}_{MRSSE}$ and ${\bar{v}}_{MRSSE}$, respectively, where $\lambda_{\left(E\right)}=\frac{1}{2n}$.

Infinite inspection surveys, the enhancement for the estimates of the objective parameter *μ*_*y*_ can be achieved by utilizing supplementary information, for instance, through the ratio, product and regression methods of estimation. Along with this heading, some authors have stretched out a piece of these outcomes to MRSS. In the following, we present a concise review of certain important contributions.

When there is a significant degree of linear relationship exists between the subject variable *Y* and the supplementary variable *X*, and the mean and quartiles of *X* are known in advance, [12] developed two mean ratio type estimators under MRSS design:

$$\begin{array}{c} {\bar{y}}_{q1}=\left(\frac{\mu_{x}+q_{1}}{{\bar{x}}_{MRSS}+q_{1}}\right){\bar{y}}_{MRSS} \end{array}$$

$$\begin{array}{c} {\bar{y}}_{q3}=\left(\frac{\mu_{x}+q_{3}}{{\bar{x}}_{MRSS}+q_{3}}\right){\bar{y}}_{MRSS}, \end{array}$$

where *μ*_*x*_ is the population mean of the random variable *X* and (*q*_1_, *q*_3_) represent the upper and lower quartiles of the supplementary variable *X*. These estimators can be refortified as

$$\begin{array}{c} {\bar{y}}_{qk(j)}={\bar{y}}_{MRSS(j)}\left(\frac{\mu_{x}+q_{k}}{{\bar{x}}_{MRSS(j)}+q_{k}}\right), \end{array}$$ (1)

where *j* = (*E*, *O*) represents the odd and even sample selection. Further, (*k* = 1, 3) denotes the first and third quartiles, respectively. The Bias and mean square error (MSE) expressions for order *n*^−1^ of ${\bar{y}}_{qk(j)}$ are

$$\begin{array}{c} Bias\left({\bar{y}}_{qk(j)}\right)≅\{\begin{array}{c} \text{if}\;n\;\text{is}\;\text{odd}\\ \frac{\theta \lambda_{\left(O\right)}\mu_{y}}{\mu_{x}}[\frac{\theta }{\mu_{x}}\sigma_{x(\frac{1+n}{2})}^{2}-\frac{1}{\mu_{y}}\sigma_{xy[\frac{1+n}{2}]}]\\ \text{if}\;n\;\text{is}\;\text{even}\\ \frac{\theta \lambda_{\left(E\right)}\mu_{y}}{\mu_{x}}[\frac{\theta }{\mu_{x}}\left(\sigma_{x(\frac{n}{2})}^{2}+\sigma_{x(\frac{2+n}{2})}^{2}\right)-\frac{1}{\mu_{y}}\left(\sigma_{xy[\frac{n}{2}]}+\sigma_{xy[\frac{2+n}{2}]}\right)] \end{array} \end{array}$$

$$\begin{array}{c} MSE\left({\bar{y}}_{qk(j)}\right)≅\{\begin{array}{c} \text{if}\;n\;\text{is}\;\text{odd}\\ \theta \lambda_{\left(O\right)}\mu_{y}[\theta_{1}^{2}\sigma_{x(\frac{1+n}{2})}^{2}+\sigma_{y[\frac{1+n}{2}]}^{2}-2\theta_{1}\sigma_{xy[\frac{1+n}{2}]}]\\ \text{if}\;n\;\text{is}\;\text{even}\\ \theta \lambda_{\left(E\right)}\mu_{y}[\theta_{1}^{2}\left(\sigma_{x(\frac{n}{2})}^{2}+\sigma_{x(\frac{2+n}{2})}^{2}\right)+\left(\sigma_{y[\frac{n}{2}]}^{2}+\sigma_{y[\frac{2+n}{2}]}^{2}\right)-2\theta_{1}\left(\sigma_{xy[\frac{n}{2}]}+\sigma_{xy[\frac{2+n}{2}]}\right)] \end{array} \end{array}$$ (2)

where $\theta =\frac{a\mu_{x}}{a\mu_{x}+b},$ for (*a* = 1, *b* = *q*_*k*_) and $\theta_{1}=\frac{\mu_{y}}{\left(\mu_{x}+q_{k}\right)}$.

The ratio estimators determined in [12] are most suitable for the positive degree of linear relationship, i.e., (*ρ*_*xy*(*j*)_ > 0). Similarly, for the negative degree of a linear relationship, i.e., (*ρ*_*xy*(*j*)_ < 0), the product versions of these estimators can be developed by taking the reciprocal of ratio type component. To defeat the constraint related to the indication of *ρ*_*xy*(*j*)_, [18] developed a usual regression-estimator under MRSS with their MSE expression as given by

$$\begin{array}{c} {\bar{y}}_{rg(j)}={\bar{y}}_{MRSS(j)}+b_{yx(j)}\left(\mu_{x}-{\bar{x}}_{MRSS(j)}\right) \end{array}$$ (3)

$$\begin{array}{c} MSE\left({\bar{y}}_{rg(j)}\right)≅\{\begin{array}{cc} \lambda_{\left(O\right)}\sigma_{y[\frac{1+n}{2}]}^{2}\left(1-\rho_{xy[\frac{1+n}{2}]}^{2}\right) & \text{if}\;n\;\text{is}\;\text{odd}\\ \lambda_{\left(E\right)}\left(\sigma_{y[\frac{n}{2}]}^{2}+\sigma_{y[\frac{2+n}{2}]}^{2}\right)\left(1-\left(\rho_{xy[\frac{1+n}{2}]}^{2}+\rho_{xy[\frac{2+n}{2}]}^{2}\right)\right) & \text{if}\;n\;\text{is}\;\text{even,} \end{array} \end{array}$$ (4)

where *b*_*xy*(*j*)_ is the regression coefficient. [19] constructed a difference type estimator based on two tuning parameters, known as generalized regression estimator, under SRS design. By merging the idea of [12, 18, 19] also introduced a difference type estimators under MRSS as follows:

$$\begin{array}{c} ({\bar{y}}_{Nk(j)}=\left[k_{1(j)}{\bar{y}}_{MRSS(j)}+k_{2(j)}\left(\mu_{x}-{\bar{x}}_{MRSS(j)}\right)\right]\;\frac{\mu_{x}+q_{k}}{{\bar{x}}_{MRSS(j)}+q_{k}}), \end{array}$$ (5)

where *k*_1(*j*)_ and *k*_2(*j*)_ are wisely chosen constants. The bias and MSE expressions are determined by

$$\begin{array}{c} Bias\left({\bar{y}}_{Nk(j)}\right)≅\{\begin{array}{c} \text{if}\;n\;\text{is}\;\text{odd}\\ \left(k_{1(O)}-1\right)\mu_{y}-\lambda_{\left(O\right)}k_{1(O)}\frac{\theta }{\mu_{x}}\sigma_{xy[\frac{1+n}{2}]}+\left(k_{2(O)}\mu_{x}\theta +\theta^{2}k_{1(O)}\mu_{y}\right)\frac{\lambda_{\left(O\right)}}{\mu_{x}^{2}}\sigma_{x(\frac{1+n}{2})}^{2}\\ \text{if}\;n\;\text{is}\;\text{even}\\ \left(k_{1(E)}-1\right)\mu_{y}-\lambda_{\left(E\right)}k_{1(E)}\frac{\theta }{\mu_{x}}\left(\sigma_{xy[\frac{n}{2}]}+\sigma_{xy[\frac{2+n}{2}]}\right)\\ +\left(k_{2(E)}\mu_{x}\theta +\theta^{2}k_{1(E)}\mu_{y}\right)\frac{\lambda_{\left(E\right)}}{\mu_{x}^{2}}\left(\sigma_{x(\frac{n}{2})}^{2}+\sigma_{x(\frac{2+n}{2})}^{2}\right) \end{array} \end{array}$$ (6)

$$\begin{array}{c} MSE_{min}\left({\bar{y}}_{Nk(j)}\right)≅\{\begin{array}{c} \text{if}\;n\;\text{is}\;\text{odd}\\ \left(1-\frac{\theta^{2}\lambda_{\left(O\right)}}{\mu_{x}^{2}}\sigma_{x(\frac{1+n}{2})}^{2}\right)\times \frac{MSE({\bar{y}}_{rg(O)})}{1-\frac{\theta^{2}\lambda_{\left(O\right)}}{\mu_{x}^{2}}\sigma_{x(\frac{1+n}{2})}^{2}+\frac{1}{\mu_{y}^{2}}MSE\left({\bar{y}}_{rg(O)}\right)}\\ \text{if}\;n\;\text{is}\;\text{even}\\ (1-\frac{\theta^{2}\lambda_{\left(E\right)}}{\mu_{x}^{2}}\left(\sigma_{x(\frac{n}{2})}^{2}+\sigma_{x(\frac{2+n}{2})}^{2}\right))\times \frac{MSE({\bar{y}}_{rg(E)})}{1-\frac{\theta^{2}\lambda_{\left(E\right)}}{\mu_{x}^{2}}\left(\sigma_{x(\frac{n}{2})}^{2}+\sigma_{x(\frac{2+n}{2})}^{2}\right)+\frac{1}{\mu_{y}^{2}}MSE\left({\bar{y}}_{rg(E)}\right)} \end{array} \end{array}$$ (7)

The optimum values of *k*_1(*j*)_ and *k*_2(*j*)_ are given by

$$\begin{array}{c} k_{1}^{opt}≅\{\begin{array}{c} \text{if}\;n\;\text{is}\;\text{odd}\\ \frac{1-\frac{\theta^{2}\lambda_{\left(O\right)}}{\mu_{x}^{2}}\sigma_{x(\frac{1+n}{2})}^{2}}{1-\frac{\theta^{2}\lambda_{\left(O\right)}}{\mu_{x}^{2}}\sigma_{x(\frac{1+n}{2})}^{2}+\frac{1}{\mu_{y}^{2}}MSE\left({\bar{y}}_{rg(O)}\right)}\\ \text{if}\;n\;\text{is}\;\text{even}\\ \frac{1-\frac{\theta^{2}\lambda_{\left(E\right)}}{\mu_{x}^{2}}\left(\sigma_{x(\frac{n}{2})}^{2}+\sigma_{x(\frac{2+n}{2})}^{2}\right)}{1-\frac{\theta^{2}\lambda_{\left(E\right)}}{2n\mu_{x}^{2}}\left(\sigma_{x(\frac{n}{2})}^{2}+\sigma_{x(\frac{2+n}{2})}^{2}\right)+\frac{1}{\mu_{y}^{2}}MSE\left({\bar{y}}_{rg(E)}\right)} \end{array} \end{array}$$ (8)

$$\begin{array}{c} k_{2}^{opt}≅\{\begin{array}{c} \text{if}\;n\;\text{is}\;\text{odd}\\ \frac{\mu_{y}}{\mu_{x}}[\theta +\frac{\left\{\frac{1}{\mu_{y}\mu_{x}}\sigma_{xy[\frac{1+n}{2}]}-2\theta \frac{1}{\mu_{x}^{2}}\sigma_{x(\frac{1+n}{2})}^{2}\}\{1-\frac{\theta^{2}\lambda_{\left(O\right)}}{\mu_{x}^{2}}\sigma_{x(\frac{1+n}{2})}^{2}\right\}}{\frac{1}{\mu_{x}^{2}}\sigma_{x(\frac{1+n}{2})}^{2}\{1-\frac{\theta^{2}\lambda_{\left(O\right)}}{\mu_{x}^{2}}\sigma_{x(\frac{1+n}{2})}^{2}+\frac{1}{\mu_{y}^{2}}MSE({\bar{y}}_{rg(O)}\}}]\\ \text{if}\;n\;\text{is}\;\text{even}\\ \frac{\mu_{y}}{\mu_{x}}[\theta +\frac{\left\{\frac{1}{\mu_{y}\mu_{x}}\left(\sigma_{xy[\frac{n}{2}]}+\sigma_{xy[\frac{2+n}{2}]}\right)-2\theta \frac{1}{\mu_{x}^{2}}\left(\sigma_{x(\frac{n}{2})}^{2}+\sigma_{x(\frac{2+n}{2})}^{2}\right)\}\{1-\frac{\theta^{2}\lambda_{\left(E\right)}}{\mu_{x}^{2}}\left(\sigma_{x(\frac{n}{2})}^{2}+\sigma_{x(\frac{2+n}{2})}^{2}\right)\right\}}{\frac{1}{\mu_{x}^{2}}\left(\sigma_{x(\frac{n}{2})}^{2}+\sigma_{x(\frac{2+n}{2})}^{2}\right)\{1-\frac{\theta^{2}\lambda_{\left(E\right)}}{\mu_{x}^{2}}\left(\sigma_{x(\frac{n}{2})}^{2}+\sigma_{x(\frac{2+n}{2})}^{2}\right)+\frac{1}{\mu_{y}^{2}}MSE\left({\bar{y}}_{rg(E)}\right)\}}] \end{array} \end{array}$$ (9)

[20] considered the ratio and product parts of the usual ratio and product estimator in exponential form and hence obtained a more enhanced estimates of the population parameter. These exponential estimators perform well according to their constraints related to indications, i.e., (*ρ*_*xy*(*j*)_ > 0) and (*ρ*_*xy*(*j*)_ < 0), for ratio and product type estimators, respectively. Taking inspiration from [20] and ${\bar{y}}_{Nk(j)}$, [18] also developed exponential type estimators under MRSS as follows:

$$\begin{array}{c} {\bar{y}}_{Kk(j)}=\left[w_{1(j)}{\bar{y}}_{MRSS(j)}+w_{2(j)}{\left(\frac{{\bar{x}}_{MRSS(j)}}{\mu_{x}}\right)}^{\alpha }\right]exp\left(\frac{\mu_{x}-{\bar{x}}_{MRSS(j)}}{\mu_{x}-{\bar{x}}_{MRSS(j)}+2q_{k}}\right) \end{array}$$ (10)

The bias and MSE expressions are as given below:

$$\begin{array}{c} Bias\left({\bar{y}}_{Kk(j)}\right)≅\{\begin{array}{c} \text{if}\;n\;\text{is}\;\text{odd}\\ w_{1(O)}\mu_{y}-\mu_{y}+w_{2(O)}-\frac{\theta \lambda_{\left(O\right)}w_{1(O)}}{2\mu_{x}}\sigma_{xy[\frac{1+n}{2}]}\\ +(\frac{w_{2(O)}}{2}\left(-\theta \alpha +\frac{3}{4}\theta^{2}+\alpha \left(\alpha -1\right)\right)\\ +\frac{3}{8}w_{1(O)}\theta^{2}\mu_{y})\frac{\lambda_{\left(O\right)}}{\mu_{x}^{2}}\sigma_{x(\frac{1+n}{2})}^{2}\\ \text{if}\;n\;\text{is}\;\text{even}\\ w_{1(E)}\mu_{y}-\mu_{y}+w_{2(E)}-\frac{\theta w_{1(E)}\lambda_{\left(E\right)}}{2\mu_{x}}\left(\sigma_{xy[\frac{n}{2}]}+\sigma_{xy[\frac{2+n}{2}]}\right)\\ +(\frac{w_{2(E)}}{2}\left(-\theta \alpha +\frac{3}{4}\theta^{2}+\alpha \left(\alpha -1\right)\right)\\ +\frac{3}{8}w_{1(E)}\theta^{2}\mu_{y})\frac{\lambda_{\left(E\right)}}{\mu_{x}^{2}}\left(\sigma_{x(\frac{n}{2})}^{2}+\sigma_{x(\frac{2+n}{2})}^{2}\right) \end{array} \end{array}$$ (11)

$$\begin{array}{c} MSE\left({\bar{y}}_{Kk(j)}\right)=\mu_{y}^{2}+w_{1(j)}^{2}\mu_{y}^{2}+A_{1(j)}+w_{2(j)}^{2}B_{1(j)}+w_{1(j)}\mu_{y}^{2}+D_{1(j)}+w_{2(j)}\mu_{y}+T_{1(j)} \end{array}$$ (12)

where

$$\begin{array}{c} A_{1(j)}=1+E\left(\varepsilon_{0(j)}^{2}\right)+\theta^{2}E\left(\varepsilon_{1(j)}^{2}\right)-2\theta E\left(\varepsilon_{0(j)}\varepsilon_{1(j)}\right),\;B_{1(j)}=1+\left(\alpha^{2}+\theta^{2}+\alpha \left(\alpha -1\right)-2\alpha \theta \right)E\left(\varepsilon_{1(j)}^{2}\right), \end{array}$$

$$\begin{array}{c} D_{1(j)}=-2+\theta E\left(\varepsilon_{0(j)}\varepsilon_{1(j)}\right)-\frac{3}{4}\theta^{2}E\left(\varepsilon_{1(j)}^{2}\right),\;G_{1(j)}=-2+\left(\theta \alpha -\alpha \left(\alpha -1\right)-\frac{3}{4}\theta^{2}\right)E\left(\varepsilon_{1(j)}^{2}\right), \end{array}$$

$$\begin{array}{c} F_{1(j)}=2+\left(\alpha \left(\alpha -1\right)-2\alpha \theta +2\theta^{2}\right)E\left(\varepsilon_{1(j)}^{2}\right)+2\left(\alpha -\theta \right)E\left(\varepsilon_{0(j)}\varepsilon_{1(j)}\right) \end{array}$$

$$\begin{array}{c} T_{1(j)}=G_{1(j)}+w_{1(j)}w_{2(j)}\mu_{y}F_{1(j)}. \end{array}$$

By substituting $w_{1(j)}^{opt}$ and $w_{2(j)}^{opt}$ in $MSE({\bar{y}}_{Kk(M)})$, we get minimum MSE, given by

$$\begin{array}{c} MSE_{min}\left({\bar{y}}_{Kk(j)}\right)=\mu_{y}^{2}\left[1-\frac{B_{1(j)}D_{1(j)}^{2}+A_{1(j)}G_{1(j)}^{2}-D_{1(j)}F_{1(j)}G_{1(j)}}{4B_{1(j)}A_{1(j)}-F_{1(j)}^{2}}\right] \end{array}$$ (13)

where

$$\begin{array}{c} w_{1(j)}^{opt}=\frac{F_{1(j)}G_{\left(j\right)}-2B_{1(j)}D_{1(j)}}{4B_{1(j)}A_{1(j)}-F_{1(j)}^{2}}, \end{array}$$ (14)

$$\begin{array}{c} w_{2(j)}^{opt}=\mu_{y}\frac{D_{1(j)}F_{1(j)}-2A_{1(j)}G_{1(j)}}{4B_{1(j)}A_{\left(j\right)}-F_{1(j)}^{2}}. \end{array}$$ (15)

## 2 Materials and methods

The reasonable utilization of the supplementary information helps in expanding the exactness of an estimator both at the structuring stage as well as at the estimation stage, see [21–33]. In social, monetary, and regular studies, the total supplementary information is frequently accessible to the inspected outline. The basic concept is when there exists an adequate measure of the relationship between (*X*, *Y*); the rank and second raw moments are also connected (correlated) with the variate of interest. Consequently, the ranked supplementary variable *V* (which is based on the ranks of *X*) can be treated as a type of supplementary variable. Similarly, the second raw moment auxiliary variable *Z* (which is based on the second raw moment of *X*) can be treated as another supplementary variable. Hence, this three-fold supplementary information can help us in expanding the proficiency of the estimators. In view of these thoughts, we propose three new estimators of the finite population mean in the upcoming sub-sections of the current section.

### 2.1 First proposed estimator

Taking motivation from [12, 18] and utilizing two out of three defined forms of auxiliary information (*X*, *Z*), we propose the following estimator as

$$\begin{array}{c} {\bar{y}}_{P1}=\left[k_{1(j)}{\bar{y}}_{MRSS(j)}+k_{2(j)}\left(\mu_{x}-{\bar{x}}_{MRSS(j)}\right)+k_{3(j)}\left(\mu_{z}-{\bar{z}}_{MRSS(j)}\right)\right]\text{exp}\;\left(\frac{a(\mu_{x}-{\bar{x}}_{MRSS(j)})}{a(\mu_{x}+{\bar{x}}_{MRSS(j)})+2b}\right) \end{array}$$ (16)

To obtain the bias and the MSE, we define

$$\begin{array}{c} \varepsilon_{0(j)}=\frac{{\bar{y}}_{MRSS(j)}-\mu_{y}}{\mu_{y}},\;\varepsilon_{1(j)}=\frac{{\bar{x}}_{MRSS(j)}-\mu_{x}}{\mu_{x}},\;\varepsilon_{2(j)}=\frac{{\bar{z}}_{MRSS(j)}-\mu_{z}}{\mu_{z}}.\; \end{array}$$

If the sample size *n* is odd, we can write

$$\begin{array}{c} E\left(\varepsilon_{0(O)}^{2}\right)=\frac{\lambda_{\left(O\right)}}{\mu_{y}^{2}}\sigma_{y[\frac{1+n}{2}]}^{2},\;E\left(\varepsilon_{1(O)}^{2}\right)=\frac{\lambda_{\left(O\right)}}{\mu_{x}^{2}}\sigma_{x(\frac{1+n}{2})}^{2},\;E\left(\varepsilon_{2(O)}^{2}\right)=\frac{\lambda_{\left(O\right)}}{\mu_{z}^{2}}\sigma_{z[\frac{1+n}{2}]}^{2},\; \end{array}$$

$$\begin{array}{c} E\left(\varepsilon_{0(O)}\varepsilon_{1(O)}\right)=\frac{\lambda_{\left(O\right)}}{\mu_{x}\mu_{y}}\sigma_{xy[\frac{1+n}{2}]},\;E\left(\varepsilon_{0(O)}\varepsilon_{2(O)}\right)=\frac{\lambda_{\left(O\right)}}{\mu_{z}\mu_{y}}\sigma_{zy[\frac{1+n}{2}]},\;E\left(\varepsilon_{1(O)}\varepsilon_{2(O)}\right)=\frac{\lambda_{\left(O\right)}}{\mu_{z}\mu_{x}}\sigma_{zx[\frac{1+n}{2}]}.\; \end{array}$$

If the sample size *n* is even, we can write

$$\begin{array}{cc} & E\left(\varepsilon_{0(E)}^{2}\right)=\frac{\lambda_{\left(E\right)}}{\mu_{y}^{2}}\left(\sigma_{y[\frac{n}{2}]}^{2}+\sigma_{y[\frac{2+n}{2}]}^{2}\right),\;E\left(\varepsilon_{1(E)}^{2}\right)=\frac{\lambda_{\left(E\right)}}{\mu_{x}^{2}}\left(\sigma_{x(\frac{n}{2})}^{2}+\sigma_{x(\frac{2+n}{2})}^{2}\right),\\ & E\left(\varepsilon_{2(E)}^{2}\right)=\frac{\lambda_{\left(E\right)}}{\mu_{z}^{2}}\left(\sigma_{z[\frac{n}{2}]}^{2}+\sigma_{z[\frac{2+n}{2}]}^{2}\right),\;E\left(\varepsilon_{0(E)}\varepsilon_{1(E)}\right)=\frac{\lambda_{\left(E\right)}}{\mu_{x}\mu_{y}}\left(\sigma_{yx[\frac{n}{2}]}+\sigma_{yx[\frac{2+n}{2}]}\right),\\ & E\left(\varepsilon_{0(E)}\varepsilon_{2(E)}\right)=\frac{\lambda_{\left(E\right)}}{\mu_{z}\mu_{y}}\left(\sigma_{zy[\frac{n}{2}]}+\sigma_{zy[\frac{2+n}{2}]}\right),\;E\left(\varepsilon_{1(E)}\varepsilon_{2(E)}\right)=\frac{\lambda_{\left(E\right)}}{\mu_{z}\mu_{x}}\left(\sigma_{zx[\frac{n}{2}]}+\sigma_{zx[\frac{2+n}{2}]}\right). \end{array}$$

Expressing the proposed estimator ${\bar{y}}_{P1}$ in terms of *ε*’s, we have

$$\begin{array}{c} {\bar{y}}_{P1}-\mu_{y}=\left[k_{1(j)}\mu_{y}\left(1+\varepsilon_{0(j)}\right)-k_{2(j)}\mu_{x}\varepsilon_{1(j)}-k_{3(j)}\mu_{z}\varepsilon_{2(j)}\right]\left(1-\frac{1}{2}\theta \varepsilon_{1(j)}+\frac{3}{8}\theta^{2}\varepsilon_{1(j)}^{2}\right)-\mu_{y} \end{array}$$ (17)

$$\begin{array}{cc} {\bar{y}}_{P1}-\mu_{y} & =k_{1(j)}\mu_{y}+k_{1(j)}\mu_{y}\varepsilon_{0(j)}-\frac{1}{2}k_{1(j)}\mu_{y}\theta \varepsilon_{1(j)}-k_{2(j)}\mu_{x}\varepsilon_{1(j)}-k_{3(j)}\mu_{z}\varepsilon_{2(j)}\\ & -\frac{1}{2}k_{1(j)}\mu_{y}\theta \varepsilon_{0(j)}\varepsilon_{1(j)}+\frac{3}{8}k_{1(j)}\mu_{y}\theta^{2}\varepsilon_{1(j)}^{2}+\frac{1}{2}k_{2(j)}\mu_{x}\theta \varepsilon_{1(j)}^{2}+\frac{1}{2}k_{3(j)}\mu_{z}\theta \varepsilon_{1(j)}\varepsilon_{2(j)}-\mu_{y} \end{array}.$$ (18)

By applying the expectation to the above expression, we determine the theoretical expression of bias of degree *n*^−1^ as

$$\begin{array}{c} Bias\left({\bar{y}}_{P1}\right)≅\{\begin{array}{c} \text{if}\;n\;\text{is}\;\text{odd}\\ \frac{1}{8}[-8\mu_{y}+\frac{4}{\mu_{x}}\lambda_{\left(O\right)}\theta \sigma_{x(\frac{1+n}{2})}\{A_{1(O)}\}]\\ +\frac{\mu_{y}}{8}k_{1(O)}[8+\frac{1}{\mu_{x}}\lambda_{\left(O\right)}\theta \sigma_{x(\frac{1+n}{2})}\{B_{1(O)}\}]\\ \text{if}\;n\;\text{is}\;\text{even}\\ \frac{1}{8}[-8\mu_{y}+\frac{4}{\mu_{x}}\lambda_{\left(E\right)}\theta \left(\sigma_{x(\frac{n}{2})}+\sigma_{x(\frac{2+n}{2})}\right)\{A_{1(E)}\}]\\ +\frac{\mu_{y}}{8}k_{1(E)}[8+\frac{1}{\mu_{x}}\lambda_{\left(E\right)}\theta \left(\sigma_{x(\frac{n}{2})}+\sigma_{x(\frac{2+n}{2})}\right)\{B_{1(E)}\}] \end{array} \end{array}$$ (19)

where

$$\begin{array}{c} A_{1(O)}=\sigma_{x(\frac{1+n}{2})}k_{2(O)}+\sigma_{z[\frac{1+n}{2}]}k_{3(O)}\rho_{xz[\frac{1+n}{2}]} \end{array}$$

$$\begin{array}{c} A_{1(E)}=\left(\sigma_{x(\frac{n}{2})}+\sigma_{x(\frac{2+n}{2})}\right)k_{2(E)}+\left(\sigma_{z[\frac{n}{2}]}+\sigma_{z[\frac{2+n}{2}]}\right)k_{3(E)}\left(\rho_{xz[\frac{n}{2}]}+\rho_{xz[\frac{2+n}{2}]}\right). \end{array}$$

$$\begin{array}{c} B_{1(O)}=\frac{3}{\mu_{x}}\theta \sigma_{x(\frac{1+n}{2})}-\frac{4}{\mu_{y}}\sigma_{y[\frac{1+n}{2}]}\rho_{xy[\frac{1+n}{2}]} \end{array}$$

$$\begin{array}{c} B_{1(E)}=\frac{3}{\mu_{x}}\theta \left(\sigma_{x(\frac{n}{2})}+\sigma_{x(\frac{2+n}{2})}\right)-\frac{4}{\mu_{y}}\left(\sigma_{y[\frac{n}{2}]}+\sigma_{y[\frac{2+n}{2}]}\right)\left(\rho_{xy[\frac{n}{2}]}+\rho_{xy[\frac{2+n}{2}]}\right). \end{array}$$

Taking the square and the expectation of (18), we obtain the MSE expressions of ${\bar{y}}_{P1}$. After partial differentiation of the MSE, we get the optimum values of weights, i.e., *k*_1_, *k*_2_, and *k*_3_. By substituting the optimum values of weights, we determine the minimum MSE expressions. The expressions of optimum weight and the minimum MSE are as follows.

$$\begin{array}{c} k_{1}≅\{\begin{array}{c} \text{if}\;n\;\text{is}\;\text{odd}\\ \frac{\left[8-\lambda_{\left(O\right)}\theta^{2}{\left(\mu_{x}^{2}\right)}^{-1}\sigma_{x(\frac{1+n}{2})}^{2}\right]}{8\left[1+A_{2(O)}\right]}\\ \text{if}\;n\;\text{is}\;\text{even}\\ \frac{\left[8-\lambda_{\left(E\right)}\theta^{2}{\left(\mu_{x}^{2}\right)}^{-1}\left(\sigma_{x(\frac{n}{2})}^{2}+\sigma_{x(\frac{2+n}{2})}^{2}\right)\right]}{8[1+A_{2(E)}]} \end{array} \end{array}$$ (20)

where

$$\begin{array}{c} A_{2(O)}=\lambda_{\left(O\right)}{\left(\mu_{y}^{2}\right)}^{-1}\left(1-P_{\left(O\right)}^{2}\right)\sigma_{y[\frac{1+n}{2}]}^{2}\;\text{and}\;A_{2(E)}=\lambda_{\left(E\right)}{\left(\mu_{y}^{2}\right)}^{-1}\left(1-P_{\left(E\right)}^{2}\right)\left(\sigma_{y[\frac{n}{2}]}^{2}+\sigma_{y[\frac{2+n}{2}]}^{2}\right). \end{array}$$

$$\begin{array}{c} k_{2}≅\{\begin{array}{c} \text{if}\;n\;\text{is}\;\text{odd}\\ \frac{\mu_{y}[\lambda_{\left(O\right)}\theta^{3}{\left(\mu_{x}^{3}\right)}^{-1}\sigma_{x(\frac{1+n}{2})}^{3}\{-1+\rho_{xz[\frac{1+n}{2}]}^{2}\}+4R\theta {\left(\mu_{x}\right)}^{-1}\sigma_{x(\frac{1+n}{2})}]}{8\sigma_{x(\frac{1+n}{2})}\{-1+\rho_{xz[\frac{1+n}{2}]}^{2}\}\{1+A_{2(O)}\}}\\ +\frac{\mu_{y}[{\left(\mu_{y}\right)}^{-1}\sigma_{y(\frac{1+n}{2})}\{-8+\lambda_{\left(O\right)}\theta^{2}{\left(\mu_{x}^{2}\right)}^{-1}\sigma_{x(\frac{1+n}{2})}^{2}\}\{\rho_{xy[\frac{1+n}{2}]}-\rho_{xz[\frac{1+n}{2}]}\rho_{zy[\frac{1+n}{2}]}\}]}{8\sigma_{x(\frac{1+n}{2})}\{-1+\rho_{xz[\frac{1+n}{2}]}^{2}\}\{1+A_{2(O)}\}}.\\ \text{if}\;n\;\text{is}\;\text{even}\\ \frac{\mu_{y}[\lambda_{\left(E\right)}\theta^{3}{\left(\mu_{x}^{3}\right)}^{-1}\left(\sigma_{x(\frac{n}{2})}^{3}+\sigma_{x(\frac{2+n}{2})}^{3}\right)\{-1+(\rho_{xz[\frac{n}{2}]}^{2}+\rho_{xz[\frac{2+n}{2}]}^{2})\}]}{8\left(\sigma_{x(\frac{n}{2})}+\sigma_{x(\frac{2+n}{2})}\right)\{-1+(\rho_{xz[\frac{n}{2}]}^{2}+\rho_{xz[\frac{2+n}{2}]}^{2})\}\{1+A_{2(E)}\}}\\ +\frac{\mu_{y}[4\theta {\left(\mu_{x}\right)}^{-1}\left(\sigma_{x(\frac{n}{2})}+\sigma_{x(\frac{2+n}{2})}\right)\{-1+(\rho_{xz[\frac{n}{2}]}^{2}+\rho_{xz[\frac{2+n}{2}]}^{2})\}\{-1+A_{2(E)}\}]}{8\left(\sigma_{x(\frac{n}{2})}+\sigma_{x(\frac{2+n}{2})}\right)\{-1+(\rho_{xz[\frac{n}{2}]}^{2}+\rho_{xz[\frac{2+n}{2}]}^{2})\}\{1+A_{2(E)}\}}\\ +\frac{\mu_{y}[{\left(\mu_{y}\right)}^{-1}\left(\sigma_{y(\frac{n}{2})}+\sigma_{y(\frac{2+n}{2})}\right)\{-8+\lambda_{\left(E\right)}\theta^{2}{\left(\mu_{x}^{2}\right)}^{-1}\left(\sigma_{x(\frac{n}{2})}^{2}+\sigma_{x(\frac{2+n}{2})}^{2}\right)\}]}{8\left(\sigma_{x(\frac{n}{2})}+\sigma_{x(\frac{2+n}{2})}\right)\{-1+(\rho_{xz[\frac{n}{2}]}^{2}+\rho_{xz[\frac{2+n}{2}]}^{2})\}\{1+A_{2(E)}\}}\\ \times \{\left(\rho_{xy[\frac{n}{2}]}+\rho_{xy[\frac{2+n}{2}]}\right)-\left(\rho_{xz[\frac{n}{2}]}+\rho_{xz[\frac{2+n}{2}]}\right)\left(\rho_{zy[\frac{1+n}{2}]}+\rho_{zy[\frac{2+n}{2}]}\right)\}. \end{array} \end{array}$$ (21)

$$\begin{array}{c} k_{3}≅\{\begin{array}{c} \text{if}\;n\;\text{is}\;\text{odd}\\ \frac{\left[\sigma_{y[\frac{1+n}{2}]}\{8-\lambda_{\left(O\right)}\theta^{2}{\left(\mu_{x}^{2}\right)}^{-1}\sigma_{x(\frac{1+n}{2})}^{2}\}\{A_{3(O)}\}\right]}{8\sigma_{z[\frac{1+n}{2}]}\{-1+\rho_{xz[\frac{1+n}{2}]}^{2}\}\{1+A_{2(O)}\}}\\ \text{if}\;n\;\text{is}\;\text{even}\\ \frac{\left[\left(\sigma_{y[\frac{n}{2}]}+\sigma_{y[\frac{2+n}{2}]}\right)\{8-\lambda_{\left(E\right)}\theta^{2}{\left(\mu_{x}^{2}\right)}^{-1}\left(\sigma_{x(\frac{n}{2})}^{2}+\sigma_{x(\frac{2+n}{2})}^{2}\right)\}\{A_{3(E)}\}\right]}{8\left(\sigma_{z[\frac{n}{2}]}+\sigma_{z[\frac{2+n}{2}]}\right)\{-1+(\rho_{xz[\frac{n}{2}]}^{2}+\rho_{xz[\frac{2+n}{2}]}^{2})\}\{1+A_{2(E)}\}} \end{array} \end{array}$$ (22)

where

$$\begin{array}{c} A_{3(O)}=-\rho_{yz[\frac{1+n}{2}]}+\rho_{xz[\frac{1+n}{2}]}\rho_{xy[\frac{1+n}{2}]}, \end{array}$$

$$\begin{array}{c} A_{3(E)}=-\left(\rho_{yz[\frac{n}{2}]}+\rho_{yz[\frac{2+n}{2}]}\right)+\left(\rho_{xz[\frac{n}{2}]}+\rho_{xz[\frac{2+n}{2}]}\right)\left(\rho_{xy[\frac{n}{2}]}+\rho_{xy[\frac{2+n}{2}]}\right), \end{array}$$

$$\begin{array}{c} R=\{-1+\rho_{xz[\frac{1+n}{2}]}^{2}\}\{-1+A_{2(O)}\}. \end{array}$$

$$\begin{array}{c} MSE\left({\bar{y}}_{P1}\right)≅\{\begin{array}{c} \text{if}\;n\;\text{is}\;\text{odd}\\ \frac{\lambda_{\left(O\right)}\mu_{y}^{2}[\frac{64}{\mu_{y}^{2}}\sigma_{y[\frac{1+n}{2}]}^{2}\left(1-P_{\left(O\right)}^{2}\right)-\frac{1}{\mu_{x}^{4}}\lambda_{\left(O\right)}\theta^{4}\sigma_{x(\frac{1+n}{2})}^{4}-R_{1}]}{64[1+\frac{1}{\mu_{y}^{2}}\lambda_{\left(O\right)}\sigma_{y[\frac{1+n}{2}]}^{2}\left(1-P_{\left(O\right)}^{2}\right)]}\\ \text{if}\;n\;\text{is}\;\text{even}\\ \frac{\lambda_{\left(E\right)}\mu_{y}^{2}[\frac{64}{\mu_{y}^{2}}\left(\sigma_{y[\frac{n}{2}]}^{2}+\sigma_{y[\frac{2+n}{2}]}^{2}\right)\left(1-P_{\left(E\right)}^{2}\right)-\frac{1}{\mu_{x}^{4}}\lambda_{\left(E\right)}\theta^{4}\left(\sigma_{x(\frac{n}{2})}^{4}+\sigma_{x(\frac{2+n}{2})}^{4}\right)]}{64[1+\frac{1}{\mu_{y}^{2}}\lambda_{\left(E\right)}\left(\sigma_{y[\frac{n}{2}]}^{2}+\sigma_{y[\frac{2+n}{2}]}^{2}\right)\left(1-P_{\left(E\right)}^{2}\right)]}\\ -\frac{\lambda_{\left(E\right)}\mu_{y}^{2}[\frac{16}{\mu_{y}^{2}\mu_{x}^{2}}\lambda_{\left(E\right)}\theta^{2}\left(\sigma_{y[\frac{n}{2}]}^{2}+\sigma_{y[\frac{2+n}{2}]}^{2}\right)\left(\sigma_{x(\frac{n}{2})}^{2}+\sigma_{x(\frac{2+n}{2})}^{2}\right)\left(1-P_{\left(E\right)}^{2}\right)]}{64[1+\frac{1}{\mu_{y}^{2}}\lambda_{\left(E\right)}\left(\sigma_{y[\frac{n}{2}]}^{2}+\sigma_{y[\frac{2+n}{2}]}^{2}\right)\left(1-P_{\left(E\right)}^{2}\right)]} \end{array} \end{array}$$ (23)

where

$$\begin{array}{c} P_{\left(O\right)}^{2}=\frac{\rho_{xy[\frac{1+n}{2}]}^{2}+\rho_{zy[\frac{1+n}{2}]}^{2}-2\rho_{xy[\frac{1+n}{2}]}\rho_{zy[\frac{1+n}{2}]}\rho_{xz[\frac{1+n}{2}]}}{1-\rho_{xz[\frac{1+n}{2}]}^{2}}, \end{array}$$

$$\begin{array}{c} P_{\left(E\right)}^{2}=\frac{\left(\rho_{xy[\frac{n}{2}]}^{2}+\rho_{xy[\frac{2+n}{2}]}^{2}\right)+\left(\rho_{zy[\frac{n}{2}]}^{2}+\rho_{zy[\frac{2+n}{2}]}^{2}\right)-2R_{2}\left(\rho_{xy[\frac{n}{2}]}+\rho_{xy[\frac{2+n}{2}]}\right)}{1-(\rho_{xz[\frac{n}{2}]}^{2}+\rho_{xz[\frac{2+n}{2}]}^{2})}. \end{array}$$

$$\begin{array}{c} R_{1}=\frac{16}{\mu_{y}^{2}\mu_{x}^{2}}\lambda_{\left(O\right)}\theta^{2}\sigma_{y[\frac{1+n}{2}]}^{2}\sigma_{x(\frac{1+n}{2})}^{2}\left(1-P_{\left(O\right)}^{2}\right) \end{array}$$

$$\begin{array}{c} R_{2}=\left(\rho_{zy[\frac{n}{2}]}+\rho_{zy[\frac{2+n}{2}]}\right)\left(\rho_{xz[\frac{n}{2}]}+\rho_{xz[\frac{2+n}{2}]}\right). \end{array}$$

### 2.2 Second proposed estimator

Taking the motivation from ${\bar{y}}_{P1}$ and utilizing two out of three defined forms of auxiliary information, i.e., (*X*, *V*), we propose the following estimator.

$$\begin{array}{c} {\bar{y}}_{P2}=\left[k_{1(j)}{\bar{y}}_{MRSS(j)}+k_{2(j)}\left(\mu_{x}-{\bar{x}}_{MRSS(j)}\right)+k_{3(j)}\left(\mu_{v}-{\bar{v}}_{MRSS(j)}\right)\right]\text{exp}\left(\frac{a(\mu_{x}-{\bar{x}}_{MRSS(j)})}{a(\mu_{x}+{\bar{x}}_{MRSS(j)})+2b}\right) \end{array}$$ (24)

$$\begin{array}{c} {\bar{y}}_{P2}-\mu_{y}=\left[k_{1(j)}\mu_{y}\left(1+\varepsilon_{0(j)}\right)-k_{2(j)}\mu_{x}\varepsilon_{1(j)}-k_{3(j)}\mu_{v}\varepsilon_{3(j)}\right]\left(1-\frac{1}{2}\theta \varepsilon_{1(j)}+\frac{3}{8}\theta^{2}\varepsilon_{1(j)}^{2}\right)-\mu_{y}. \end{array}$$ (25)

To obtain the bias and the MSE, we define

$$\begin{array}{c} \varepsilon_{3(j)}=\frac{{\bar{v}}_{MRSS(j)}-\mu_{v}}{\mu_{v}}.\; \end{array}$$

If the sample size *n* is odd, we have

$$\begin{array}{cc} & E\left(\varepsilon_{3(O)}^{2}\right)=\frac{\lambda_{\left(O\right)}}{\mu_{v}^{2}}\sigma_{v[\frac{1+n}{2}]}^{2},\;E\left(\varepsilon_{0(O)}\varepsilon_{3(O)}\right)=\frac{\lambda_{\left(O\right)}}{\mu_{v}\mu_{y}}\sigma_{vy[\frac{1+n}{2}]},\;E\left(\varepsilon_{1(O)}\varepsilon_{3(O)}\right)=\frac{\lambda_{\left(O\right)}}{\mu_{v}\mu_{x}}\sigma_{vx[\frac{1+n}{2}]}. \end{array}$$

If the sample size *n* is even, we write

$$\begin{array}{cc} & E\left(\varepsilon_{3(E)}^{2}\right)=\frac{\lambda_{\left(E\right)}}{\mu_{v}^{2}}\left(\sigma_{v[\frac{n}{2}]}^{2}+\sigma_{v[\frac{2+n}{2}]}^{2}\right),\;E\left(\varepsilon_{0(E)}\varepsilon_{3(E)}\right)=\frac{\lambda_{\left(E\right)}}{\mu_{v}\mu_{y}}\left(\sigma_{vy[\frac{n}{2}]}+\sigma_{vy[\frac{2+n}{2}]}\right),\\ & E\left(\varepsilon_{1(E)}\varepsilon_{3(E)}\right)=\frac{\lambda_{\left(E\right)}}{\mu_{v}\mu_{x}}\left(\sigma_{vx[\frac{n}{2}]}+\sigma_{vx[\frac{2+n}{2}]}\right). \end{array}$$

Accordingly, the bias and minimum MSE can be calculated from the expressions provided in previous section, by replacing the second raw moment auxiliary variable *Z* with the corresponding ranked supplementary variable *V*. Hence, we have

$$\begin{array}{c} Bias\left({\bar{y}}_{P2}\right)≅\{\begin{array}{c} \text{if}\;n\;\text{is}\;\text{odd}\\ \frac{1}{8}[-8\mu_{y}+\frac{4}{\mu_{x}}\lambda_{\left(O\right)}\theta \sigma_{x(\frac{1+n}{2})}\{A_{3(O)}\}]\\ +\frac{\mu_{y}}{8}k_{1(O)}[8+\frac{1}{\mu_{x}}\lambda_{\left(O\right)}\theta \sigma_{x(\frac{1+n}{2})}\{B_{3(O)}\}]\\ \text{if}\;n\;\text{is}\;\text{even}\\ \frac{1}{8}[-8\mu_{y}+\frac{4}{\mu_{x}}\lambda_{\left(E\right)}\theta \left(\sigma_{x(\frac{n}{2})}+\sigma_{x(\frac{2+n}{2})}\right)\{A_{3(E)}\}]\\ +\frac{\mu_{y}}{8}k_{1(E)}[8+\frac{1}{\mu_{x}}\lambda_{\left(E\right)}\theta \left(\sigma_{x(\frac{n}{2})}+\sigma_{x(\frac{2+n}{2})}\right)\{B_{3(E)}\}] \end{array} \end{array}$$ (26)

where

$$\begin{array}{c} A_{3(O)}=\sigma_{x(\frac{1+n}{2})}k_{2(O)}+\sigma_{v[\frac{1+n}{2}]}k_{3(O)}\rho_{xv[\frac{1+n}{2}]} \end{array}$$

$$\begin{array}{c} A_{3(E)}=\left(\sigma_{x(\frac{n}{2})}+\sigma_{x(\frac{2+n}{2})}\right)k_{2(E)}+\left(\sigma_{v[\frac{n}{2}]}+\sigma_{v[\frac{2+n}{2}]}\right)k_{3(E)}\left(\rho_{xv[\frac{n}{2}]}+\rho_{xv[\frac{2+n}{2}]}\right). \end{array}$$

$$\begin{array}{c} B_{3(O)}=\frac{3}{\mu_{x}}\theta \sigma_{x(\frac{1+n}{2})}-\frac{4}{\mu_{y}}\sigma_{y[\frac{1+n}{2}]}\rho_{xy[\frac{1+n}{2}]} \end{array}$$

$$\begin{array}{c} B_{3(E)}=\frac{3}{\mu_{x}}\theta \left(\sigma_{x(\frac{n}{2})}+\sigma_{x(\frac{2+n}{2})}\right)-\frac{4}{\mu_{y}}\left(\sigma_{y[\frac{n}{2}]}+\sigma_{y[\frac{2+n}{2}]}\right)\left(\rho_{xy[\frac{n}{2}]}+\rho_{xy[\frac{2+n}{2}]}\right). \end{array}$$

$$\begin{array}{c} MSE\left({\bar{y}}_{P2}\right)≅\{\begin{array}{c} \text{if}\;n\;\text{is}\;\text{odd}\\ \frac{\lambda_{\left(O\right)}\mu_{y}^{2}[\frac{64}{\mu_{y}^{2}}\sigma_{y[\frac{1+n}{2}]}^{2}\left(1-P_{\left(O\right)}^{2}\right)-\frac{1}{\mu_{x}^{4}}\lambda_{\left(O\right)}\theta^{4}\sigma_{x(\frac{1+n}{2})}^{4}-R_{3}]}{64[1+\frac{1}{\mu_{y}^{2}}\lambda_{\left(O\right)}\sigma_{y[\frac{1+n}{2}]}^{2}\left(1-P_{\left(O\right)}^{2}\right)]}\\ \text{if}\;n\;\text{is}\;\text{even}\\ \frac{\lambda_{\left(E\right)}\mu_{y}^{2}[\frac{64}{\mu_{y}^{2}}\left(\sigma_{y[\frac{n}{2}]}^{2}+\sigma_{y[\frac{2+n}{2}]}^{2}\right)\left(1-P_{\left(E\right)}^{2}\right)-\frac{1}{\mu_{x}^{4}}\lambda_{\left(E\right)}\theta^{4}\left(\sigma_{x(\frac{n}{2})}^{4}+\sigma_{x(\frac{2+n}{2})}^{4}\right)]}{64[1+\frac{1}{\mu_{y}^{2}}\lambda_{\left(E\right)}\left(\sigma_{y[\frac{n}{2}]}^{2}+\sigma_{y[\frac{2+n}{2}]}^{2}\right)\left(1-P_{\left(E\right)}^{2}\right)]}\\ -\frac{\lambda_{\left(E\right)}\mu_{y}^{2}[\frac{16}{\mu_{y}^{2}\mu_{x}^{2}}\lambda_{\left(E\right)}\theta^{2}\left(\sigma_{y[\frac{n}{2}]}^{2}+\sigma_{y[\frac{2+n}{2}]}^{2}\right)\left(\sigma_{x(\frac{n}{2})}^{2}+\sigma_{x(\frac{2+n}{2})}^{2}\right)\left(1-P_{\left(E\right)}^{2}\right)]}{64[1+\frac{1}{\mu_{y}^{2}}\lambda_{\left(E\right)}\left(\sigma_{y[\frac{n}{2}]}^{2}+\sigma_{y[\frac{2+n}{2}]}^{2}\right)\left(1-P_{\left(E\right)}^{2}\right)]} \end{array} \end{array}$$ (27)

where

$$\begin{array}{c} R_{3}=\frac{16}{\mu_{y}^{2}\mu_{x}^{2}}\lambda_{\left(O\right)}\theta^{2}\sigma_{y[\frac{1+n}{2}]}^{2}\sigma_{x(\frac{1+n}{2})}^{2}\left(1-P_{\left(O\right)}^{2}\right). \end{array}$$

For abbreviation, we intentionally skip the expressions of $k_{1},k_{2},k_{3},P_{\left(O\right)}^{2}$ and $P_{\left(E\right)}^{2}$. Interested readers may easily get the mathematical expressions of these quantities by simply replacing *z* with *v*, regarding the previous sub-section.

### 2.3 Third proposed estimator

Taking motivation from ${\bar{y}}_{P1}$ and ${\bar{y}}_{P2}$ and utilizing all the three defined forms of auxiliary information, i.e., (*X*, *V*, *Z*), we propose the following estimator

$$\begin{array}{c} {\bar{y}}_{P3}=\left[k_{1(j)}{\bar{y}}_{MRSS(j)}+k_{2(j)}\left(\mu_{x}-{\bar{x}}_{MRSS(j)}\right)+k_{3(j)}\left(\mu_{v}-{\bar{v}}_{MRSS(j)}\right)+k_{4(j)}\left(\mu_{z}-{\bar{z}}_{MRSS(j)}\right)\right]\times G_{p} \end{array}$$ (28)

where

$$\begin{array}{c} G_{p}=\text{exp}\;\left(\frac{a(\mu_{x}-{\bar{x}}_{MRSS(j)})}{a(\mu_{x}+{\bar{x}}_{MRSS(j)})+2b}\right). \end{array}$$ (29)

$$\begin{array}{c} {\bar{y}}_{P3}-\mu_{y}=[k_{1(j)}\mu_{y}\left(1+\varepsilon_{0(j)}\right)-k_{2(j)}\mu_{x}\varepsilon_{1(j)}-k_{3(j)}\mu_{v}\varepsilon_{3(j)}-R_{4}, \end{array}$$ (30)

where

$$\begin{array}{c} R_{4}=k_{4(j)}\mu_{z}\varepsilon_{2(j)}]\left(1-\frac{1}{2}\theta \varepsilon_{1(j)}+\frac{3}{8}\theta^{2}\varepsilon_{1(j)}^{2}\right)-\mu_{y}. \end{array}$$ (31)

The bias of ${\bar{y}}_{P3}$ is

$$\begin{array}{c} Bias\left({\bar{y}}_{P3}\right)≅\{\begin{array}{c} \text{if}\;n\;\text{is}\;\text{odd}\\ \mu_{y}\left(k_{1(O)}-1\right)+\frac{3}{8}\lambda_{\left(O\right)}\theta^{2}\mu_{y}k_{1(O)}{\left(\mu_{x}^{2}\right)}^{-1}\sigma_{x(\frac{1+n}{2})}^{2}\\ -\frac{1}{2}\lambda_{\left(O\right)}\theta k_{1(O)}{\left(\mu_{x}\right)}^{-1}\sigma_{x(\frac{1+n}{2})}\sigma_{y[\frac{1+n}{2}]}\rho_{yx[\frac{1+n}{2}]}\\ +\frac{1}{2}\lambda_{\left(O\right)}\theta \mu_{x}k_{2(O)}{\left(\mu_{x}^{2}\right)}^{-1}\sigma_{x(\frac{1+n}{2})}^{2}+\frac{1}{2}\lambda_{\left(O\right)}\theta k_{3(O)}{\left(\mu_{x}\right)}^{-1}\sigma_{x(\frac{1+n}{2})}\sigma_{v[\frac{1+n}{2}]}\rho_{vx[\frac{1+n}{2}]}\\ +\frac{1}{2}\lambda_{\left(O\right)}\theta k_{4(O)}{\left(\mu_{x}\right)}^{-1}\sigma_{x(\frac{1+n}{2})}\sigma_{z[\frac{1+n}{2}]}\rho_{zx[\frac{1+n}{2}]}\\ \text{if}\;n\;\text{is}\;\text{even}\\ \mu_{y}\left(k_{1(E)}-1\right)+\frac{3}{8}\lambda_{\left(E\right)}\theta^{2}\mu_{y}k_{1(E)}{\left(\mu_{x}^{2}\right)}^{-1}\left(\sigma_{x(\frac{n}{2})}^{2}+\sigma_{x(\frac{2+n}{2})}^{2}\right)\\ -\frac{1}{2}\lambda_{\left(E\right)}\theta k_{1(E)}{\left(\mu_{x}\right)}^{-1}\left(\sigma_{x(\frac{n}{2})}+\sigma_{x(\frac{2+n}{2})}\right)\left(\sigma_{y[\frac{n}{2}]}+\sigma_{y[\frac{2+n}{2}]}\right)\left(\rho_{xy[\frac{n}{2}]}+\rho_{xy[\frac{2+n}{2}]}\right)\\ +\frac{1}{2}\lambda_{\left(E\right)}\theta \mu_{x}k_{2(E)}{\left(\mu_{x}^{2}\right)}^{-1}\left(\sigma_{x(\frac{n}{2})}^{2}+\sigma_{x(\frac{2+n}{2})}^{2}\right)\\ +\frac{1}{2}\lambda_{\left(E\right)}\theta k_{3(E)}{\left(\mu_{x}\right)}^{-1}\left(\sigma_{x(\frac{n}{2})}+\sigma_{x(\frac{2+n}{2})}\right)\left(\sigma_{v[\frac{n}{2}]}+\sigma_{v[\frac{2+n}{2}]}\right)\left(\rho_{vx[\frac{n}{2}]}+\rho_{vx[\frac{2+n}{2}]}\right)\\ +\frac{1}{2}\lambda_{\left(E\right)}\theta k_{4(E)}{\left(\mu_{x}\right)}^{-1}\left(\sigma_{x(\frac{n}{2})}+\sigma_{x(\frac{2+n}{2})}\right)\left(\sigma_{z[\frac{n}{2}]}+\sigma_{z[\frac{2+n}{2}]}\right)\left(\rho_{zx[\frac{n}{2}]}+\rho_{zx[\frac{2+n}{2}]}\right) \end{array} \end{array}$$ (32)

Taking the square and the expectation of (30), we get the MSE expressions of ${\bar{y}}_{P3}$. Then, by partial differentiation of the MSE, we determine optimum values of weights, i.e., *k*_1_, *k*_2_, *k*_3_, and *k*_4_. By substituting the optimum values of weights, we obtain the minimum MSE expressions. The optimum weights and minimum MSE are as follows.

$$\begin{array}{c} k_{1}≅\{\begin{array}{c} \text{if}\;n\;\text{is}\;\text{odd}\\ \frac{\left[\{t_{1(O)}-1\}\{\lambda_{\left(O\right)}\theta^{2}{\left(\mu_{x}^{2}\right)}^{-1}\sigma_{x(\frac{1+n}{2})}^{2}-8\}\right]}{8\left[\lambda_{\left(O\right)}{\left(\mu_{y}^{2}\right)}^{-1}\sigma_{y(\frac{1+n}{2})}^{2}\{t_{3(O)}+t_{4(O)}+t_{2(O)}+1\}+1+t_{1(O)}\right]}\\ \text{if}\;n\;\text{is}\;\text{even}\\ \frac{\left[\{t_{1(E)}-1\}\{\lambda_{\left(E\right)}\theta^{2}{\left(\mu_{x}^{2}\right)}^{-1}\left(\sigma_{x(\frac{n}{2})}^{2}+\sigma_{x(\frac{2+n}{2})}^{2}\right)-8\}\right]}{8\left[\lambda_{\left(E\right)}{\left(\mu_{y}^{2}\right)}^{-1}\left(\sigma_{y[\frac{n}{2}]}^{2}+\sigma_{y[\frac{2+n}{2}]}^{2}\right)\{t_{3(E)}+t_{4(E)}+t_{2(E)}+1\}+1+t_{1(E)}\right]} \end{array} \end{array}$$ (33)

$$\begin{array}{c} k_{2}≅\{\begin{array}{c} \text{if}\;n\;\text{is}\;\text{odd}\\ \frac{-4\mu_{y}[-\frac{1}{4}\theta^{3}\lambda_{\left(O\right)}{\left(\mu_{x}^{3}\right)}^{-1}\sigma_{x(\frac{1+n}{2})}^{3}\left(t_{1(O)}-1\right)-\frac{1}{4}\theta^{2}\lambda_{\left(O\right)}{\left(\mu_{y}\right)}^{-1}\sigma_{y[\frac{1+n}{2}]}{\left(\mu_{x}^{2}\right)}^{-1}\sigma_{x(\frac{1+n}{2})}^{2}t_{5(O)}]}{\sigma_{x(\frac{1+n}{2})}[\lambda_{\left(O\right)}{\left(\mu_{y}^{2}\right)}^{-1}\sigma_{y[\frac{1+n}{2}]}^{2}\{t_{3(O)}+t_{4(O)}+t_{2(O)}+1\}+1+t_{1(O)}]}\\ +\frac{-4\mu_{y}[\lambda_{\left(O\right)}{\left(\mu_{y}^{2}\right)}^{-1}\sigma_{y[\frac{1+n}{2}]}^{2}\left(t_{3(O)}+t_{4(O)}-t_{2(O)}+1\right)+\theta {\left(\mu_{x}\right)}^{-1}\sigma_{x(\frac{1+n}{2})}+2{\left(\mu_{y}\right)}^{-1}\sigma_{y[\frac{1+n}{2}]}t_{5(O)}]}{\sigma_{x(\frac{1+n}{2})}[\lambda_{\left(O\right)}{\left(\mu_{y}^{2}\right)}^{-1}\sigma_{y[\frac{1+n}{2}]}^{2}\{t_{3(O)}+t_{4(O)}+t_{2(O)}+1\}+1+t_{1(O)}]}\\ \text{if}\;n\;\text{is}\;\text{even}\\ \frac{-4\mu_{y}[-\frac{1}{4}\theta^{3}\lambda_{\left(E\right)}{\left(\mu_{x}^{3}\right)}^{-1}\left(\sigma_{x(\frac{n}{2})}^{3}+\sigma_{x(\frac{2+n}{2})}^{3}\right)\left(t_{1(E)}-1\right)-R_{5}]}{\left(\sigma_{x(\frac{n}{2})}+\sigma_{x(\frac{2+n}{2})}\right)[\lambda_{\left(E\right)}{\left(\mu_{y}^{2}\right)}^{-1}\left(\sigma_{y[\frac{n}{2}]}^{2}+\sigma_{y[\frac{2+n}{2}]}^{2}\right)\{t_{3(E)}+t_{4(E)}+t_{2(E)}+1\}+1+t_{1(E)}]}\\ +\frac{-4\mu_{y}[R_{6}+\theta {\left(\mu_{x}\right)}^{-1}\left(\sigma_{x(\frac{n}{2})}+\sigma_{x(\frac{2+n}{2})}\right)+2{\left(\mu_{y}\right)}^{-1}\left(\sigma_{y[\frac{n}{2}]}+\sigma_{y[\frac{2+n}{2}]}\right)t_{5(E)}]}{\left(\sigma_{x(\frac{n}{2})}+\sigma_{x(\frac{2+n}{2})}\right)[\lambda_{\left(E\right)}{\left(\mu_{y}^{2}\right)}^{-1}\left(\sigma_{y[\frac{n}{2}]}^{2}+\sigma_{y[\frac{2+n}{2}]}^{2}\right)\{t_{3(E)}+t_{4(E)}+t_{2(E)}+1\}+1+t_{1(E)}]} \end{array} \end{array}$$ (34)

$$\begin{array}{c} k_{3}≅\{\begin{array}{c} \text{if}\;n\;\text{is}\;\text{odd}\\ \frac{\left[-\sigma_{y[\frac{1+n}{2}]}t_{6(O)}\{\lambda_{\left(O\right)}\theta^{2}{\left(\mu_{x}^{2}\right)}^{-1}\sigma_{x(\frac{1+n}{2})}^{2}-8\}\right]}{8\sigma_{v[\frac{1+n}{2}]}\left[\lambda_{\left(O\right)}{\left(\mu_{y}^{2}\right)}^{-1}\sigma_{y[\frac{1+n}{2}]}^{2}\{t_{3(O)}+t_{4(O)}+t_{2(O)}+1\}+1+t_{1(O)}\right]}\\ \text{if}\;n\;\text{is}\;\text{even}\\ \frac{\left[-\left(\sigma_{y[\frac{n}{2}]}+\sigma_{y[\frac{2+n}{2}]}\right)t_{6(E)}\{\lambda_{\left(E\right)}\theta^{2}{\left(\mu_{x}^{2}\right)}^{-1}\left(\sigma_{x(\frac{n}{2})}^{2}+\sigma_{x(\frac{2+n}{2})}^{2}\right)-8\}\right]}{8\left(\sigma_{v[\frac{n}{2}]}+\sigma_{v[\frac{2+n}{2}]}\right)\left[\lambda_{\left(E\right)}{\left(\mu_{y}^{2}\right)}^{-1}\left(\sigma_{y[\frac{n}{2}]}^{2}+\sigma_{y[\frac{2+n}{2}]}^{2}\right)\{t_{3(E)}+t_{4(E)}+t_{2(E)}+1\}+1+t_{1(E)}\right]} \end{array} \end{array}$$ (35)

$$\begin{array}{c} k_{4}≅\{\begin{array}{c} \text{if}\;n\;\text{is}\;\text{odd}\\ \frac{\left[-\sigma_{y[\frac{1+n}{2}]}t_{7(O)}\{\lambda_{\left(O\right)}\theta^{2}{\left(\mu_{x}^{2}\right)}^{-1}\sigma_{x(\frac{1+n}{2})}^{2}-8\}\right]}{8\sigma_{z[\frac{1+n}{2}]}\left[\lambda_{\left(O\right)}{\left(\mu_{y}^{2}\right)}^{-1}\sigma_{y[\frac{1+n}{2}]}^{2}\{t_{3(O)}+t_{4(O)}+t_{2(O)}+1\}+1+t_{1(O)}\right]}\\ \text{if}\;n\;\text{is}\;\text{even}\\ \frac{\left[-\left(\sigma_{y[\frac{n}{2}]}+\sigma_{y[\frac{2+n}{2}]}\right)t_{7(E)}\{\lambda_{\left(E\right)}\theta^{2}{\left(\mu_{x}^{2}\right)}^{-1}\left(\sigma_{x(\frac{n}{2})}^{2}+\sigma_{x(\frac{2+n}{2})}^{2}\right)-8\}\right]}{8\left(\sigma_{z[\frac{n}{2}]}+\sigma_{z[\frac{2+n}{2}]}\right)\left[\lambda_{\left(E\right)}{\left(\mu_{y}^{2}\right)}^{-1}\left(\sigma_{y[\frac{n}{2}]}^{2}+\sigma_{y[\frac{2+n}{2}]}^{2}\right)\{t_{3(E)}+t_{4(E)}+t_{2(E)}+1\}+1+t_{1(E)}\right]} \end{array} \end{array}$$ (36)

$$\begin{array}{c} MSE\left({\bar{y}}_{P3}\right)≅\{\begin{array}{c} \text{if}\;n\;\text{is}\;\text{odd}\\ \frac{\lambda_{\left(O\right)}\mu_{y}^{2}[R_{7}-\frac{1}{16}\lambda_{\left(O\right)}\theta^{4}{\left(\mu_{x}^{2}\right)}^{-1}\sigma_{x(\frac{1+n}{2})}^{2}\{t_{1(O)}-1\}]}{4[t_{1(O)}-1-\lambda_{\left(O\right)}{\left(\mu_{y}^{2}\right)}^{-1}\sigma_{y[\frac{1+n}{2}]}^{2}\{t_{3(O)}+t_{4(O)}-t_{2(O)}+1\}]}\\ \text{if}\;n\;\text{is}\;\text{even}\\ \frac{\lambda_{\left(E\right)}\mu_{y}^{2}[\{t_{3(E)}+t_{4(E)}-t_{2(E)}+1\}R_{8}{\left(\mu_{y}^{2}\right)}^{-1}\left(\sigma_{y[\frac{n}{2}]}^{2}+\sigma_{y[\frac{2+n}{2}]}^{2}\right)]}{4[t_{1(E)}-1-\lambda_{\left(E\right)}{\left(\mu_{y}^{2}\right)}^{-1}\left(\sigma_{y[\frac{n}{2}]}^{2}+\sigma_{y[\frac{2+n}{2}]}^{2}\right)\{t_{3(E)}+t_{4(E)}-t_{2(E)}+1\}]}\\ +\frac{\lambda_{\left(E\right)}\mu_{y}^{2}[-\frac{1}{16}\lambda_{\left(E\right)}\theta^{4}{\left(\mu_{x}^{2}\right)}^{-1}\left(\sigma_{x(\frac{n}{2})}^{2}+\sigma_{x(\frac{2+n}{2})}^{2}\right)\{t_{1(E)}-1\}]}{4[t_{1(E)}-1-\lambda_{\left(E\right)}{\left(\mu_{y}^{2}\right)}^{-1}\left(\sigma_{y[\frac{n}{2}]}^{2}+\sigma_{y[\frac{2+n}{2}]}^{2}\right)\{t_{3(E)}+t_{4(E)}-t_{2(E)}+1\}]}. \end{array} \end{array}$$ (37)

where

$$\begin{array}{c} R_{5}=\frac{1}{4}\theta^{2}\lambda_{\left(E\right)}{\left(\mu_{y}\right)}^{-1}\left(\sigma_{y[\frac{n}{2}]}+\sigma_{y[\frac{2+n}{2}]}\right){\left(\mu_{x}^{2}\right)}^{-1}\left(\sigma_{x(\frac{n}{2})}^{2}+\sigma_{x(\frac{2+n}{2})}^{2}\right)t_{5(E)} \end{array}$$

$$\begin{array}{c} R_{6}=\lambda_{\left(E\right)}{\left(\mu_{y}^{2}\right)}^{-1}\left(\sigma_{y[\frac{n}{2}]}^{2}+\sigma_{y[\frac{2+n}{2}]}^{2}\right)\left(t_{3(E)}+t_{4(E)}-t_{2(E)}+1\right) \end{array}$$

$$\begin{array}{c} R_{7}=\{t_{3(O)}+t_{4(O)}-t_{2(O)}+1\}\{\lambda_{\left(O\right)}\theta^{2}{\left(\mu_{x}^{4}\right)}^{-1}\sigma_{x(\frac{1+n}{2})}^{4}-4\}{\left(\mu_{y}^{2}\right)}^{-1}\sigma_{y[\frac{1+n}{2}]}^{2} \end{array}$$

$$\begin{array}{c} R_{8}=\{\lambda_{\left(E\right)}\theta^{2}{\left(\mu_{x}^{4}\right)}^{-1}\left(\sigma_{x(\frac{n}{2})}^{4}+\sigma_{x(\frac{2+n}{2})}^{4}\right)-4\}. \end{array}$$

As we see that optimum values of *k*_1_, *k*_2_, *k*_3_, *k*_4_ and the minimum MSE contain the notations *t*_1(*O*)_, *t*_2(*O*)_, …, *t*_7(*O*)_ for odd sample size and *t*_1(*E*)_, *t*_2(*E*)_, …, *t*_7(*E*)_ for even sample size. The detailed expressions of these notations *t*_1(*j*)_, *t*_2(*j*)_, …, *t*_7(*j*)_, are as follows.

$$\begin{array}{c} t_{1(j)}≅\{\begin{array}{c} \text{if}\;n\;\text{is}\;\text{odd}\\ \rho_{vx[\frac{1+n}{2}]}^{2}+\rho_{zx[\frac{1+n}{2}]}^{2}+\rho_{vz[\frac{1+n}{2}]}^{2}-2\rho_{vx[\frac{1+n}{2}]}\rho_{zx[\frac{1+n}{2}]}\rho_{vz[\frac{1+n}{2}]}\\ \text{if}\;n\;\text{is}\;\text{even}\\ \left(\rho_{vx[\frac{n}{2}]}^{2}+\rho_{vx[\frac{2+n}{2}]}^{2}\right)+\left(\rho_{zx[\frac{n}{2}]}^{2}+\rho_{zx[\frac{2+n}{2}]}^{2}\right)+\left(\rho_{vz[\frac{n}{2}]}^{2}+\rho_{vz[\frac{2+n}{2}]}^{2}\right)\\ -2\left(\rho_{vx[\frac{n}{2}]}+\rho_{vx[\frac{2+n}{2}]}\right)\left(\rho_{zx[\frac{n}{2}]}+\rho_{zx[\frac{2+n}{2}]}\right)\left(\rho_{vz[\frac{n}{2}]}+\rho_{vz[\frac{2+n}{2}]}\right) \end{array} \end{array}$$

$$\begin{array}{c} t_{2(j)}≅\{\begin{array}{c} \text{if}\;n\;\text{is}\;\text{odd}\\ \rho_{yx[\frac{1+n}{2}]}^{2}+\rho_{yv[\frac{1+n}{2}]}^{2}+\rho_{yz[\frac{1+n}{2}]}^{2}-2\rho_{yx[\frac{1+n}{2}]}\rho_{yv[\frac{1+n}{2}]}\rho_{xz[\frac{1+n}{2}]}\\ \text{if}\;n\;\text{is}\;\text{even}\\ \left(\rho_{yx[\frac{n}{2}]}^{2}+\rho_{yx[\frac{2+n}{2}]}^{2}\right)+\left(\rho_{yv[\frac{n}{2}]}^{2}+\rho_{yv[\frac{2+n}{2}]}^{2}\right)+\left(\rho_{yz[\frac{n}{2}]}^{2}+\rho_{yz[\frac{2+n}{2}]}^{2}\right)\\ -2\left(\rho_{yx[\frac{n}{2}]}+\rho_{yx[\frac{2+n}{2}]}\right)\left(\rho_{yv[\frac{n}{2}]}+\rho_{yv[\frac{2+n}{2}]}\right)\left(\rho_{xz[\frac{n}{2}]}+\rho_{xz[\frac{2+n}{2}]}\right) \end{array} \end{array}$$

$$\begin{array}{c} t_{3(j)}≅\{\begin{array}{c} \text{if}\;n\;\text{is}\;\text{odd}\\ \left\{\rho_{yx[\frac{1+n}{2}]}^{2}-1\}\rho_{vz[\frac{1+n}{2}]}^{2}+2\rho_{vx[\frac{1+n}{2}]}\{-\rho_{yx[\frac{1+n}{2}]}\rho_{yz[\frac{1+n}{2}]}+\rho_{xz[\frac{1+n}{2}]}\right\}\\ +4\rho_{vx[\frac{1+n}{2}]}\rho_{yv[\frac{1+n}{2}]}\rho_{vz[\frac{1+n}{2}]}\{-\rho_{yx[\frac{1+n}{2}]}\rho_{xz[\frac{1+n}{2}]}+\rho_{yz[\frac{1+n}{2}]}\}\\ \text{if}\;n\;\text{is}\;\text{even}\\ \{(\rho_{yx[\frac{n}{2}]}^{2}+\rho_{yx[\frac{2+n}{2}]}^{2})-1\}\left(\rho_{vz[\frac{n}{2}]}^{2}+\rho_{vz[\frac{2+n}{2}]}^{2}\right)\\ +2\left(\rho_{vx[\frac{n}{2}]}+\rho_{vx[\frac{2+n}{2}]}\right)\{-\left(\rho_{yx[\frac{n}{2}]}+\rho_{yx[\frac{2+n}{2}]}\right)\left(\rho_{yz[\frac{n}{2}]}+\rho_{yz[\frac{2+n}{2}]}\right)+\left(\rho_{xz[\frac{n}{2}]}+\rho_{xz[\frac{2+n}{2}]}\right)\}\\ +4\left(\rho_{vx[\frac{n}{2}]}+\rho_{vx[\frac{2+n}{2}]}\right)\left(\rho_{yv[\frac{n}{2}]}+\rho_{yv[\frac{2+n}{2}]}\right)\left(\rho_{vz[\frac{n}{2}]}+\rho_{vz[\frac{2+n}{2}]}\right)\\ \times \{-\left(\rho_{yx[\frac{n}{2}]}+\rho_{yx[\frac{2+n}{2}]}\right)\left(\rho_{xz[\frac{n}{2}]}+\rho_{xz[\frac{2+n}{2}]}\right)+\left(\rho_{yz[\frac{n}{2}]}+\rho_{yz[\frac{2+n}{2}]}\right)\} \end{array} \end{array}$$

$$\begin{array}{c} t_{4(j)}≅\{\begin{array}{c} \text{if}\;n\;\text{is}\;\text{odd}\\ \{\rho_{yz[\frac{1+n}{2}]}^{2}-1\}\rho_{vx[\frac{1+n}{2}]}^{2}+\{\rho_{vy[\frac{1+n}{2}]}^{2}-1\}\rho_{zx[\frac{1+n}{2}]}^{2}\\ -2\rho_{vy[\frac{1+n}{2}]}\rho_{vx[\frac{1+n}{2}]}\{\rho_{yz[\frac{1+n}{2}]}\rho_{xz[\frac{1+n}{2}]}-\rho_{yx[\frac{1+n}{2}]}\}\\ \text{if}\;n\;\text{is}\;\text{even}\\ \{(\rho_{yz[\frac{n}{2}]}^{2}+\rho_{yz[\frac{2+n}{2}]}^{2})-1\}\left(\rho_{vx[\frac{n}{2}]}^{2}+\rho_{vx[\frac{2+n}{2}]}^{2}\right)\\ +\{(\rho_{vy[\frac{n}{2}]}^{2}+\rho_{vy[\frac{2+n}{2}]}^{2})-1\}\left(\rho_{zx[\frac{n}{2}]}^{2}+\rho_{zx[\frac{2+n}{2}]}^{2}\right)\\ -2\left(\rho_{vy[\frac{n}{2}]}+\rho_{vy[\frac{2+n}{2}]}\right)\left(\rho_{vx[\frac{n}{2}]}+\rho_{vx[\frac{2+n}{2}]}\right)\\ \times \{\left(\rho_{yz[\frac{n}{2}]}+\rho_{yz[\frac{2+n}{2}]}\right)\left(\rho_{xz[\frac{n}{2}]}+\rho_{xz[\frac{2+n}{2}]}\right)-\left(\rho_{yx[\frac{n}{2}]}+\rho_{yx[\frac{2+n}{2}]}\right)\} \end{array} \end{array}$$

$$\begin{array}{c} t_{5(j)}≅\{\begin{array}{c} \text{if}\;n\;\text{is}\;\text{odd}\\ -\rho_{yx[\frac{1+n}{2}]}\rho_{vz[\frac{1+n}{2}]}^{2}+\{\rho_{yz[\frac{1+n}{2}]}\rho_{vx[\frac{1+n}{2}]}+\rho_{vy[\frac{1+n}{2}]}\rho_{zx[\frac{1+n}{2}]}\}\rho_{vz[\frac{1+n}{2}]}\\ -\rho_{zy[\frac{1+n}{2}]}\rho_{zx[\frac{1+n}{2}]}-\rho_{vy[\frac{1+n}{2}]}\rho_{vx[\frac{1+n}{2}]}+\rho_{yx[\frac{1+n}{2}]}\\ \text{if}\;n\;\text{is}\;\text{even}\\ -\left(\rho_{yx[\frac{n}{2}]}+\rho_{yx[\frac{2+n}{2}]}\right)\left(\rho_{vz[\frac{n}{2}]}^{2}+\rho_{vz[\frac{2+n}{2}]}^{2}\right)\\ +\{\left(\rho_{yz[\frac{n}{2}]}+\rho_{yz[\frac{2+n}{2}]}\right)\left(\rho_{vx[\frac{n}{2}]}+\rho_{vx[\frac{2+n}{2}]}\right)+\left(\rho_{vy[\frac{n}{2}]}+\rho_{vy[\frac{2+n}{2}]}\right)\left(\rho_{zx[\frac{n}{2}]}+\rho_{zx[\frac{2+n}{2}]}\right)\}\\ \times \left(\rho_{vz[\frac{n}{2}]}+\rho_{vz[\frac{2+n}{2}]}\right)\\ -\left(\rho_{zy[\frac{n}{2}]}+\rho_{zy[\frac{2+n}{2}]}\right)\left(\rho_{zx[\frac{n}{2}]}+\rho_{zx[\frac{2+n}{2}]}\right)-\left(\rho_{vy[\frac{n}{2}]}+\rho_{vy[\frac{2+n}{2}]}\right)\left(\rho_{vx[\frac{n}{2}]}+\rho_{vx[\frac{2+n}{2}]}\right)\\ +\left(\rho_{yx[\frac{n}{2}]}+\rho_{yx[\frac{2+n}{2}]}\right) \end{array} \end{array}$$

$$\begin{array}{c} t_{6(j)}≅\{\begin{array}{c} \text{if}\;n\;\text{is}\;\text{odd}\\ \rho_{yx[\frac{1+n}{2}]}\rho_{zx[\frac{1+n}{2}]}\rho_{vz[\frac{1+n}{2}]}+\rho_{yz[\frac{1+n}{2}]}\rho_{vx[\frac{1+n}{2}]}\rho_{zx[\frac{1+n}{2}]}-\rho_{vy[\frac{1+n}{2}]}\rho_{zx[\frac{1+n}{2}]}^{2}\\ -\rho_{zy[\frac{1+n}{2}]}\rho_{vz[\frac{1+n}{2}]}-\rho_{yx[\frac{1+n}{2}]}\rho_{vx[\frac{1+n}{2}]}+\rho_{vy[\frac{1+n}{2}]}\\ \text{if}\;n\;\text{is}\;\text{even}\\ \left(\rho_{yx[\frac{n}{2}]}+\rho_{yx[\frac{2+n}{2}]}\right)\left(\rho_{zx[\frac{n}{2}]}+\rho_{zx[\frac{2+n}{2}]}\right)\left(\rho_{vz[\frac{n}{2}]}+\rho_{vz[\frac{2+n}{2}]}\right)\\ +\left(\rho_{yz[\frac{n}{2}]}+\rho_{yz[\frac{2+n}{2}]}\right)\left(\rho_{vx[\frac{n}{2}]}+\rho_{vx[\frac{2+n}{2}]}\right)\left(\rho_{zx[\frac{n}{2}]}+\rho_{zx[\frac{2+n}{2}]}\right)\\ -\left(\rho_{vy[\frac{n}{2}]}+\rho_{vy[\frac{2+n}{2}]}\right)\left(\rho_{zx[\frac{n}{2}]}^{2}+\rho_{zx[\frac{2+n}{2}]}^{2}\right)\\ -\left(\rho_{zy[\frac{n}{2}]}+\rho_{zy[\frac{2+n}{2}]}\right)\left(\rho_{vz[\frac{n}{2}]}+\rho_{vz[\frac{2+n}{2}]}\right)\\ -\left(\rho_{yx[\frac{n}{2}]}+\rho_{yx[\frac{2+n}{2}]}\right)\left(\rho_{vx[\frac{n}{2}]}+\rho_{vx[\frac{2+n}{2}]}\right)\\ +\left(\rho_{vy[\frac{n}{2}]}+\rho_{vy[\frac{2+n}{2}]}\right) \end{array} \end{array}$$

$$\begin{array}{c} t_{7(j)}≅\{\begin{array}{c} \text{if}\;n\;\text{is}\;\text{odd}\\ \rho_{yx[\frac{1+n}{2}]}\rho_{vx[\frac{1+n}{2}]}\rho_{vz[\frac{1+n}{2}]}+\rho_{vy[\frac{1+n}{2}]}\rho_{vx[\frac{1+n}{2}]}\rho_{zx[\frac{1+n}{2}]}-\rho_{zy[\frac{1+n}{2}]}\rho_{vx[\frac{1+n}{2}]}^{2}\\ -\rho_{vy[\frac{1+n}{2}]}\rho_{vz[\frac{1+n}{2}]}-\rho_{yx[\frac{1+n}{2}]}\rho_{zx[\frac{1+n}{2}]}+\rho_{zy[\frac{1+n}{2}]}\\ \text{if}\;n\;\text{is}\;\text{even}\\ \left(\rho_{yx[\frac{n}{2}]}+\rho_{yx[\frac{2+n}{2}]}\right)\left(\rho_{vx[\frac{n}{2}]}+\rho_{vx[\frac{2+n}{2}]}\right)\left(\rho_{vz[\frac{n}{2}]}+\rho_{vz[\frac{2+n}{2}]}\right)\\ +\left(\rho_{vy[\frac{n}{2}]}+\rho_{vy[\frac{2+n}{2}]}\right)\left(\rho_{vx[\frac{n}{2}]}+\rho_{vx[\frac{2+n}{2}]}\right)\left(\rho_{zx[\frac{n}{2}]}+\rho_{zx[\frac{2+n}{2}]}\right)\\ -\left(\rho_{zy[\frac{n}{2}]}+\rho_{zy[\frac{2+n}{2}]}\right)\left(\rho_{vx[\frac{n}{2}]}^{2}+\rho_{vx[\frac{2+n}{2}]}^{2}\right)\\ -\left(\rho_{vy[\frac{n}{2}]}+\rho_{vy[\frac{2+n}{2}]}\right)\left(\rho_{vz[\frac{n}{2}]}+\rho_{vz[\frac{2+n}{2}]}\right)\\ -\left(\rho_{yx[\frac{n}{2}]}+\rho_{yx[\frac{2+n}{2}]}\right)\left(\rho_{zx[\frac{n}{2}]}+\rho_{zx[\frac{2+n}{2}]}\right)\\ +\left(\rho_{zy[\frac{n}{2}]}+\rho_{zy[\frac{2+n}{2}]}\right). \end{array} \end{array}$$

## 3 Results and discussion

### 3.1 Simulation study

While assessing the performance of the new recommended estimators, it is traditional to infer the MSE based conditions under which an estimator is more efficient than the others. However, these MSE based conditions are generally hard to confirm. Therefore, we have abstained intentionally from these conditions dependent on the MSE expressions and lean towards various numerical reenactment tests (simulation experiments), in the current Section. In the next Section, we will also assess the performance on behalf of the real life data set.

We perform the simulation study for evaluating the features of new estimators, i.e., $\left({\bar{y}}_{P1},{\bar{y}}_{P2},{\bar{y}}_{P3}\right)$ over existing ones, i.e., $({\bar{y}}_{q1(j)},{\bar{y}}_{q3(j)},{\bar{y}}_{rg(j)},{\bar{y}}_{N1(j)},{\bar{y}}_{N3(j)},,{\bar{y}}_{Kk1(j)},{\bar{y}}_{Kk3(j)})$ in MRSS. In this section, we conduct re-enactment tests to investigate the properties of the proposed estimators. In reenactment tests, we generate a population of size 10000 from a bivariate normal distribution $N(\mu_{x}=2,\mu_{y}=4,\sigma_{x}^{2}=1,\sigma_{y}^{2}=1,\rho_{xy})$ where, we assumed values of *ρ*_*xy*_ = {±0.95, ±0.75, ±0.50, ±0.35, ±0.25, ±0.15}. For more details, interested readers may be referred to [18]. It is worth mentioning that only one form of the supplementary information, i.e., the supplementary variable *X*, generated from the bi-variate normal distribution. However, the remaining two forms of the supplementary information, i.e., (*V*, *Z*), are developed by taking ranks and square of *X*.

We consider two situations: First one is based on the true descriptives of population while the later one is based on the estimated descriptives of population.

### 3.2 Simulation with known population descriptives

In order to determine the percentage relative mean square error (PRMSE) of the concerned estimators, we consider both even and odd types of sample sizes, in light of the preliminaries defined in Section 2. We consider *n* = 3, 5 as odd and *n* = 4, 6 as even sample size. It is worth mentioning that each mentioned MRSS sample of size even/odd, selected 5000 times and the averaged MSE calculated. The PRMSE is calculated as:

$$PRMSE\left(\hat{\theta }\right)=\frac{MSE(\hat{\theta })}{\mu_{y}^{2}}\times 100.$$

In the current sub-section, a simulation design is based on the assumption that complete supplementary information is available. Thus, the true population parameters of supplementary information are considered here.

### 3.3 Simulation with unknown population descriptives

The configuration of the simulation portrayed in the previous sub-section depends on the supposition that all descriptives of the population regarding both the supplementary and the subject variables, and shown in the equations of the estimators, are known, except for *μ*_*y*_. Without doubt, in spite of the fact that this circumstance has a hypothetical natural worth, its utility might be faulty in genuine examinations where some of the descriptives of supplementary variate are obscure. Thus, we have additionally investigated the adequacy of the proposed estimators under the reasonable circumstances where the previously mentioned population descriptives are obscure and should be assessed on the grounds that no dependable guess is accessible from past information, specialist advice or a pilot study. In such a condition, estimates of the objective parameters are influenced by an additional wellspring of variability; their conduct might be non-unique in relation to the situation where the population descriptives are thought to be given. To reveal insight into the matter, we address the issue of assessing the impact on the productivity of the reviewed and proposed estimators when all population descriptives, except for *μx*, are evaluated on the premise of various samples with different sizes. It is worth mentioning that the estimated optimum weights of the considered estimators are utilized for this particular situation.

### 3.4 Real life application

In this section, the estimators addressed in the previous section are assessed using real life data. Similar frame work of the previous section is adapted here. The data belongs to the production of corn and extension of agriculture land. Unit of production is “quintal” and considered as the target variable. However, the unit of extension of agriculture land is “hectares” and considered as an auxiliary variable, (Source: Istat—Italian Statistical Institute). The data set contain *N* = 101 observations with *ρ*_*xy*_ = 0.91, *μ*_*y*_ = 96.48, *μ*_*x*_ = 171.93, *σ*_*x*_ = 99.71, *σ*_*y*_ = 44.38, *q*_1_ = 80.61, *q*_2_ = 157.52, *q*_3_ = 352.13 and skewness = 0.4335.

### 3.5 Interpretation of numerical results

The analysis of the designed based simulation and the real life application are given in Tables 1–5, respectively. From these Tables, we notice that all proposed estimators ${\bar{y}}_{P1}-{\bar{y}}_{P3}$, beat ${\bar{y}}_{q1}$, ${\bar{y}}_{q3}$, ${\bar{y}}_{rg}$, ${\bar{y}}_{Nk1}$, ${\bar{y}}_{Nk2}$, ${\bar{y}}_{Kk1}$, ${\bar{y}}_{Kk2}$. Moreover, ${\bar{y}}_{rg}$ is always better than ${\bar{y}}_{q1}-{\bar{y}}_{q3}$. No consistent improvement is found between ${\bar{y}}_{Kk1}-{\bar{y}}_{Kk2}$. However, both of these are better compared to their reviewed counterparts ${\bar{y}}_{q1},{\bar{y}}_{q3},{\bar{y}}_{rg},{\bar{y}}_{Nk1},{\bar{y}}_{Nk2}$. We see that the proposed estimators are more effective than the reviewed counterparts.

**Table 1:** PRMSE for simulation study with *ρ*_*xy*_ = (0.15, 0.25, 0.35).

		True parameters				Estimated parameters			
	$\hat{\theta }$	*n* = 3	*n* = 4	*n* = 5	*n* = 6	*n* = 3	*n* = 4	*n* = 5	*n* = 6
(*ρ*_*xy*_ = 0.15)	${\bar{y}}_{q1}$	0.47847	0.47046	0.46607	0.46338	0.47848	0.47040	0.46601	0.46319
	${\bar{y}}_{q3}$	0.48161	0.47360	0.46921	0.46652	0.48162	0.47354	0.46915	0.46633
	${\bar{y}}_{rg}$	0.47046	0.46245	0.45806	0.45537	0.47047	0.46239	0.45800	0.45518
	${\bar{y}}_{Nk1}$	0.46448	0.45647	0.45208	0.44939	0.46449	0.45641	0.45202	0.44920
	${\bar{y}}_{Nk2}$	0.46045	0.45244	0.44805	0.44536	0.46046	0.45238	0.44799	0.44517
	${\bar{y}}_{Kk1}$	0.45707	0.44906	0.44467	0.44198	0.45708	0.44900	0.44461	0.44179
	${\bar{y}}_{Kk2}$	0.43972	0.43171	0.42732	0.42463	0.43973	0.43165	0.42726	0.42444
	${\bar{y}}_{P1}$	0.42149	0.41348	0.40909	0.40640	0.42150	0.41342	0.40903	0.40621
	${\bar{y}}_{P2}$	0.41383	0.40582	0.40143	0.39874	0.41384	0.40576	0.40137	0.39855
	${\bar{y}}_{P3}$	0.40870	0.40069	0.39630	0.39361	0.40871	0.40063	0.39624	0.39342
(*ρ*_*xy*_ = 0.25)	${\bar{y}}_{q1}$	0.39738	0.38937	0.38498	0.38229	0.39739	0.38931	0.38492	0.38210
	${\bar{y}}_{q3}$	0.40052	0.39251	0.38812	0.38543	0.40053	0.39245	0.38806	0.38524
	${\bar{y}}_{rg}$	0.38937	0.38136	0.37697	0.37428	0.38938	0.38130	0.37691	0.37409
	${\bar{y}}_{Nk1}$	0.38339	0.37538	0.37099	0.36830	0.38340	0.37532	0.37093	0.36811
	${\bar{y}}_{Nk2}$	0.37936	0.37135	0.36696	0.36427	0.37937	0.37129	0.36690	0.36408
	${\bar{y}}_{Kk1}$	0.37598	0.36797	0.36358	0.36089	0.37599	0.36791	0.36352	0.36070
	${\bar{y}}_{Kk2}$	0.35863	0.35062	0.34623	0.34354	0.35864	0.35056	0.34617	0.34335
	${\bar{y}}_{P1}$	0.34040	0.33239	0.32800	0.32531	0.34041	0.33233	0.32794	0.32512
	${\bar{y}}_{P2}$	0.33274	0.32473	0.32034	0.31765	0.33275	0.32467	0.32028	0.31746
	${\bar{y}}_{P3}$	0.32761	0.31960	0.31521	0.31252	0.32762	0.31954	0.31515	0.31233
(*ρ*_*xy*_ = 0.35)	${\bar{y}}_{q1}$	0.31629	0.30828	0.30389	0.30120	0.31630	0.30822	0.30383	0.30101
	${\bar{y}}_{q3}$	0.31943	0.31142	0.30703	0.30434	0.31944	0.31136	0.30697	0.30415
	${\bar{y}}_{rg}$	0.30828	0.30027	0.29588	0.29319	0.30829	0.30021	0.29582	0.29300
	${\bar{y}}_{Nk1}$	0.30230	0.29429	0.28990	0.28721	0.30231	0.29423	0.28984	0.28702
	${\bar{y}}_{Nk2}$	0.29827	0.29026	0.28587	0.28318	0.29828	0.29020	0.28581	0.28299
	${\bar{y}}_{Kk1}$	0.29489	0.28688	0.28249	0.27980	0.29490	0.28682	0.28243	0.27961
	${\bar{y}}_{Kk2}$	0.27754	0.26953	0.26514	0.26245	0.27755	0.26947	0.26508	0.26226
	${\bar{y}}_{P1}$	0.25931	0.25130	0.24691	0.24422	0.25932	0.25124	0.24685	0.24403
	${\bar{y}}_{P2}$	0.25165	0.24364	0.23925	0.23656	0.25166	0.24358	0.23919	0.23637
	${\bar{y}}_{P3}$	0.24652	0.23851	0.23412	0.23143	0.24653	0.23845	0.23406	0.23124

**Table 2:** PRMSE for simulation study with *ρ*_*xy*_ = (0.50, 0.75, 0.95).

		True parameters				Estimated parameters			
	$\hat{\theta }$	*n* = 3	*n* = 4	*n* = 5	*n* = 6	*n* = 3	*n* = 4	*n* = 5	*n* = 6
(*ρ*_*xy*_ = 0.50)	${\bar{y}}_{q1}$	0.23520	0.22719	0.22280	0.22011	0.23521	0.22713	0.22274	0.21992
	${\bar{y}}_{q3}$	0.23316	0.22518	0.22073	0.21800	0.23305	0.22507	0.22062	0.21780
	${\bar{y}}_{rg}$	0.23231	0.22433	0.21991	0.21722	0.23221	0.22428	0.21983	0.21701
	${\bar{y}}_{Nk1}$	0.23195	0.22397	0.21952	0.21681	0.23184	0.22386	0.21941	0.21665
	${\bar{y}}_{Nk2}$	0.23053	0.22255	0.21810	0.21541	0.23042	0.22244	0.21800	0.21523
	${\bar{y}}_{Kk1}$	0.22976	0.22178	0.21733	0.21460	0.22965	0.22167	0.21722	0.21440
	${\bar{y}}_{Kk2}$	0.22742	0.21942	0.21500	0.21231	0.22731	0.21933	0.21491	0.21213
	${\bar{y}}_{P1}$	0.21747	0.20949	0.20504	0.20231	0.21736	0.20938	0.20493	0.20211
	${\bar{y}}_{P2}$	0.20844	0.20046	0.19602	0.19331	0.20832	0.20035	0.19590	0.19312
	${\bar{y}}_{P3}$	0.19016	0.18218	0.17773	0.17500	0.19005	0.18207	0.17762	0.17480
(*ρ*_*xy*_ = 0.75)	${\bar{y}}_{q1}$	0.17924	0.17126	0.16681	0.16411	0.17913	0.17116	0.16671	0.16391
	${\bar{y}}_{q3}$	0.17596	0.16798	0.16353	0.16080	0.17585	0.16787	0.16342	0.16060
	${\bar{y}}_{rg}$	0.16813	0.16015	0.15572	0.15301	0.16804	0.16005	0.15560	0.15281
	${\bar{y}}_{Nk1}$	0.16518	0.15719	0.15275	0.15002	0.16507	0.15709	0.15264	0.14982
	${\bar{y}}_{Nk2}$	0.15313	0.14515	0.14070	0.13801	0.15302	0.14504	0.14061	0.13783
	${\bar{y}}_{Kk1}$	0.14914	0.14116	0.13671	0.13402	0.14903	0.14105	0.13660	0.13380
	${\bar{y}}_{Kk2}$	0.14516	0.13718	0.13273	0.13000	0.14505	0.13707	0.13262	0.12980
	${\bar{y}}_{P1}$	0.04517	0.03719	0.03274	0.03001	0.04506	0.03708	0.03263	0.02982
	${\bar{y}}_{P2}$	0.02513	0.01715	0.01270	0.02101	0.02502	0.01704	0.01260	0.00981
	${\bar{y}}_{P3}$	0.01616	0.00818	0.00373	0.00130	0.01605	0.00807	0.00362	0.00180
(*ρ*_*xy*_ = 0.95)	${\bar{y}}_{q1}$	0.07714	0.06916	0.06471	0.06201	0.07704	0.06908	0.06463	0.06180
	${\bar{y}}_{q3}$	0.03216	0.02418	0.01973	0.01700	0.03205	0.02407	0.01962	0.01680
	${\bar{y}}_{rg}$	0.02707	0.01909	0.01464	0.01191	0.02694	0.01898	0.01451	0.01170
	${\bar{y}}_{Nk1}$	0.02686	0.01888	0.01443	0.01170	0.02675	0.01877	0.01432	0.01150
	${\bar{y}}_{Nk2}$	0.02643	0.01846	0.01401	0.01131	0.02633	0.01835	0.01391	0.01110
	${\bar{y}}_{Kk1}$	0.02675	0.01877	0.01431	0.01161	0.02664	0.01866	0.01421	0.01139
	${\bar{y}}_{Kk2}$	0.02626	0.01828	0.01383	0.01110	0.02615	0.01817	0.01372	0.01090
	${\bar{y}}_{P1}$	0.02514	0.01716	0.01271	0.01200	0.02503	0.01705	0.01261	0.00981
	${\bar{y}}_{P2}$	0.02217	0.01419	0.00974	0.00700	0.02206	0.01408	0.00963	0.00680
	${\bar{y}}_{P3}$	0.00815	0.00017	0.00572	0.00300	0.00804	0.01006	0.00561	0.00280

**Table 3:** PRMSE for simulation study with *ρ*_*xy*_ = (−0.15, −0.25, −0.35).

		True parameters				Estimated parameters			
	$\hat{\theta }$	*n* = 3	*n* = 4	*n* = 5	*n* = 6	*n* = 3	*n* = 4	*n* = 5	*n* = 6
(*ρ*_*xy*_ = −0.15)	${\bar{y}}_{q1}$	0.55956	0.55155	0.54716	0.54447	0.55957	0.55149	0.54710	0.54428
	${\bar{y}}_{q3}$	0.56270	0.55469	0.55030	0.54761	0.56271	0.55463	0.55024	0.54742
	${\bar{y}}_{rg}$	0.55155	0.54354	0.53915	0.53646	0.55156	0.54348	0.53909	0.53627
	${\bar{y}}_{Nk1}$	0.54557	0.53756	0.53317	0.53048	0.54558	0.53750	0.53311	0.53029
	${\bar{y}}_{Nk2}$	0.54154	0.53353	0.52914	0.52645	0.54155	0.53347	0.52908	0.52626
	${\bar{y}}_{Kk1}$	0.53816	0.53015	0.52576	0.52307	0.53817	0.53009	0.52570	0.52288
	${\bar{y}}_{Kk2}$	0.52081	0.51280	0.50841	0.50572	0.52082	0.51274	0.50835	0.50553
	${\bar{y}}_{P1}$	0.50258	0.49457	0.49018	0.48749	0.50259	0.49451	0.49012	0.48730
	${\bar{y}}_{P2}$	0.49492	0.48691	0.48252	0.47983	0.49493	0.48685	0.48246	0.47964
	${\bar{y}}_{P3}$	0.48979	0.48178	0.47739	0.47470	0.48980	0.48172	0.47733	0.47451
(*ρ*_*xy*_ = −0.25)	${\bar{y}}_{q1}$	0.47847	0.47046	0.46607	0.46338	0.47848	0.47040	0.46601	0.46319
	${\bar{y}}_{q3}$	0.48161	0.47360	0.46921	0.46652	0.48162	0.47354	0.46915	0.46633
	${\bar{y}}_{rg}$	0.47046	0.46245	0.45806	0.45537	0.47047	0.46239	0.45800	0.45518
	${\bar{y}}_{Nk1}$	0.46448	0.45647	0.45208	0.44939	0.46449	0.45641	0.45202	0.44920
	${\bar{y}}_{Nk2}$	0.46045	0.45244	0.44805	0.44536	0.46046	0.45238	0.44799	0.44517
	${\bar{y}}_{Kk1}$	0.45707	0.44906	0.44467	0.44198	0.45708	0.44900	0.44461	0.44179
	${\bar{y}}_{Kk2}$	0.43972	0.43171	0.42732	0.42463	0.43973	0.43165	0.42726	0.42444
	${\bar{y}}_{P1}$	0.42149	0.41348	0.40909	0.40640	0.42150	0.41342	0.40903	0.40621
	${\bar{y}}_{P2}$	0.41383	0.40582	0.40143	0.39874	0.41384	0.40576	0.40137	0.39855
	${\bar{y}}_{P3}$	0.40870	0.40069	0.39630	0.39361	0.40871	0.40063	0.39624	0.39342
(*ρ*_*xy*_ = −0.35)	${\bar{y}}_{q1}$	0.39738	0.38937	0.38498	0.38229	0.39739	0.38931	0.38492	0.38210
	${\bar{y}}_{q3}$	0.40052	0.39251	0.38812	0.38543	0.40053	0.39245	0.38806	0.38524
	${\bar{y}}_{rg}$	0.38937	0.38136	0.37697	0.37428	0.38938	0.38130	0.37691	0.37409
	${\bar{y}}_{Nk1}$	0.38339	0.37538	0.37099	0.36830	0.38340	0.37532	0.37093	0.36811
	${\bar{y}}_{Nk2}$	0.37936	0.37135	0.36696	0.36427	0.37937	0.37129	0.36690	0.36408
	${\bar{y}}_{Kk1}$	0.37598	0.36797	0.36358	0.36089	0.37599	0.36791	0.36352	0.36070
	${\bar{y}}_{Kk2}$	0.35863	0.35062	0.34623	0.34354	0.35864	0.35056	0.34617	0.34335
	${\bar{y}}_{P1}$	0.34040	0.33239	0.32800	0.32531	0.34041	0.33233	0.32794	0.32512
	${\bar{y}}_{P2}$	0.33274	0.32473	0.32034	0.31765	0.33275	0.32467	0.32028	0.31746
	${\bar{y}}_{P3}$	0.32761	0.31960	0.31521	0.31252	0.32762	0.31954	0.31515	0.31233

**Table 4:** PRMSE for simulation study with *ρ*_*xy*_ = (−0.50, −0.75, −0.95).

		True parameters				Estimated parameters			
	$\hat{\theta }$	*n* = 3	*n* = 4	*n* = 5	*n* = 6	*n* = 3	*n* = 4	*n* = 5	*n* = 6
(*ρ*_*xy*_ = −0.50)	${\bar{y}}_{q1}$	0.31629	0.30828	0.30389	0.30120	0.31630	0.30822	0.30383	0.30101
	${\bar{y}}_{q3}$	0.31943	0.31142	0.30703	0.30434	0.31944	0.31136	0.30697	0.30415
	${\bar{y}}_{rg}$	0.30828	0.30027	0.29588	0.29319	0.30829	0.30021	0.29582	0.29300
	${\bar{y}}_{Nk1}$	0.30230	0.29429	0.28990	0.28721	0.30231	0.29423	0.28984	0.28702
	${\bar{y}}_{Nk2}$	0.29827	0.29026	0.28587	0.28318	0.29828	0.29020	0.28581	0.28299
	${\bar{y}}_{Kk1}$	0.29489	0.28688	0.28249	0.27980	0.29490	0.28682	0.28243	0.27961
	${\bar{y}}_{Kk2}$	0.27754	0.26953	0.26514	0.26245	0.27755	0.26947	0.26508	0.26226
	${\bar{y}}_{P1}$	0.25931	0.25130	0.24691	0.24422	0.25932	0.25124	0.24685	0.24403
	${\bar{y}}_{P2}$	0.25165	0.24364	0.23925	0.23656	0.25166	0.24358	0.23919	0.23637
	${\bar{y}}_{P3}$	0.24652	0.23851	0.23412	0.23143	0.24653	0.23845	0.23406	0.23124
(*ρ*_*xy*_ = −0.75)	${\bar{y}}_{q1}$	0.26033	0.25235	0.24790	0.24520	0.26022	0.25225	0.24780	0.24500
	${\bar{y}}_{q3}$	0.26347	0.25549	0.25104	0.24834	0.26336	0.25539	0.25094	0.24814
	${\bar{y}}_{rg}$	0.25232	0.24434	0.23989	0.23719	0.25221	0.24424	0.23979	0.23699
	${\bar{y}}_{Nk1}$	0.24634	0.23836	0.23391	0.23121	0.24623	0.23826	0.23381	0.23101
	${\bar{y}}_{Nk2}$	0.24231	0.23433	0.22988	0.22718	0.24220	0.23423	0.22978	0.22698
	${\bar{y}}_{Kk1}$	0.23893	0.23095	0.22650	0.22380	0.23882	0.23085	0.22640	0.22360
	${\bar{y}}_{Kk2}$	0.22158	0.21360	0.20915	0.20645	0.22147	0.21350	0.20905	0.20625
	${\bar{y}}_{P1}$	0.20335	0.19537	0.19092	0.18822	0.20324	0.19527	0.19082	0.18802
	${\bar{y}}_{P2}$	0.19569	0.18771	0.18326	0.18056	0.19558	0.18761	0.18316	0.18036
	${\bar{y}}_{P3}$	0.19056	0.18258	0.17813	0.17543	0.19045	0.18248	0.17803	0.17523
(*ρ*_*xy*_ = −0.95)	${\bar{y}}_{q1}$	0.15823	0.15025	0.14580	0.14310	0.15813	0.15017	0.14572	0.14289
	${\bar{y}}_{q3}$	0.16137	0.15339	0.14894	0.14624	0.16127	0.15331	0.14886	0.14603
	${\bar{y}}_{rg}$	0.15022	0.14224	0.13779	0.13509	0.15012	0.14216	0.13771	0.13488
	${\bar{y}}_{Nk1}$	0.14424	0.13626	0.13181	0.12911	0.14414	0.13618	0.13173	0.12890
	${\bar{y}}_{Nk2}$	0.14021	0.13223	0.12778	0.12508	0.14011	0.13215	0.12770	0.12487
	${\bar{y}}_{Kk1}$	0.13683	0.12885	0.12440	0.12170	0.13673	0.12877	0.12432	0.12149
	${\bar{y}}_{Kk2}$	0.11948	0.11150	0.10705	0.10435	0.11938	0.11142	0.10697	0.10414
	${\bar{y}}_{P1}$	0.10125	0.09327	0.08882	0.08612	0.10115	0.09319	0.08874	0.08591
	${\bar{y}}_{P2}$	0.09359	0.08561	0.08116	0.07846	0.09349	0.08553	0.08108	0.07825
	${\bar{y}}_{P3}$	0.08846	0.08048	0.07603	0.07333	0.08836	0.08040	0.07595	0.07312

**Table 5:** PRMSE for corn production estimates.

	True parameters				Estimated parameters			
$\hat{\theta }$	*n* = 3	*n* = 4	*n* = 5	*n* = 6	*n* = 3	*n* = 4	*n* = 5	*n* = 6
${\bar{y}}_{q1}$	2.5043	2.1250	1.9040	1.7021	1.6918	1.8441	2.3716	2.1374
${\bar{y}}_{q3}$	2.4873	2.1080	1.8870	1.6851	1.6748	1.8271	2.3546	2.1204
${\bar{y}}_{rg}$	2.4752	2.0959	1.8749	1.6730	1.6627	1.8150	2.3425	2.1083
${\bar{y}}_{Nk1}$	2.4741	2.0948	1.8738	1.6719	1.6616	1.8139	2.3414	2.1072
${\bar{y}}_{Nk2}$	2.5234	2.1441	1.9231	1.7212	1.7109	1.8632	2.3907	2.1565
${\bar{y}}_{Kk1}$	2.4772	2.0979	1.8769	1.6750	1.6647	1.8170	2.3445	2.1103
${\bar{y}}_{Kk2}$	2.4717	2.0924	1.8714	1.6695	1.6592	1.8115	2.3390	2.1048
${\bar{y}}_{P1}$	1.9522	1.5729	1.3519	1.1500	1.1397	1.2920	1.8195	1.5853
${\bar{y}}_{P2}$	1.9620	1.5827	1.3617	1.1598	1.1495	1.3018	1.8293	1.5951
${\bar{y}}_{P3}$	1.85093	1.47163	1.25063	1.04873	1.03843	1.19073	1.71823	1.48403

The PRMSE values decrease as the correlation coefficient values increase. Also, for fixed values of the correlation coefficient and the same estimator, the MSE values decrease as the sample size increases for both true and estimated parameters. This examination reveals the superiority of the proposed estimators ${\bar{y}}_{P1}-{\bar{y}}_{P3}$ over the reviewed estimators. Among all PRMSE estimators, computations are given in Tables 1–5, it can be noted that the ${\bar{y}}_{P3}$ is more efficient in terms of the PRMSE. These are the results for the circumstances considered in the numerical study, conducted for the purposes of the paper. Hence, we feel sure that the equivalent could hold in other settings.

## 4 Conclusion

In the current study, we have proposed some new estimators for the mean estimation of a subject variable when supplementary information is accessible. We explore the three-fold utilization of a single supplementary variable under MRSS design. Our class sums up the usual regression estimator and difference estimator of [19], due to [18], under MRSS design. We have also calculated the asymptotic theoretical properties such as the bias and minimum MSE. These properties are helpful to pay attention to an underestimation of the population parameter of the subject and the variable, i.e., *μ*_*y*_. The proposed estimators are not exhaustive but rather can go about as an obstruction against the expansion of equivalent propositions that could show up later on. The proficiency of the proposed estimator has been explored by comparing it with different reviewed estimators, based on hypothetical and practical examples. For this, numerical examinations on some genuine and artificial populations have been conducted by Monte Carlo studies. These numerical examinations show the prevalence of the proposed estimators over the existing ones. Thus, the new proposals, based on three-fold utilization of a single supplementary variable, are recommended for survey practitioners. In future studies, the proposed three fold utilization can be extended for two stages MRSS in light of [12, 18, 34] work.
